# Vascular adhesion protein-1 defines a unique subpopulation of human hematopoietic stem cells and regulates their proliferation

**DOI:** 10.1007/s00018-021-03977-6

**Published:** 2021-11-01

**Authors:** Imtiaz Iftakhar-e-Khuda, Alberto Pessia, Shuyu Zheng, Matti Kankainen, Mika Kontro, Marika Karikoski, Juha Laurila, Heidi Gerke, Sina Tadayon, Maija Hollmén, Jing Tang, Beat A. Imhof, Marko Salmi, Sirpa Jalkanen

**Affiliations:** 1 Medicity Research Laboratory, University of Turku, 20520 Turku, Finland; 2 Research Program in Systems Oncology (ONCOSYS), Faculty of Medicine, University of Helsinki, Helsinki, Finland; 3 Department of Hematology, Helsinki University Hospital, Helsinki, Finland; 4 Department of Pathology and Immunology, Centre Médical Universitaire (CMU), University of Geneva, Rue Michel-Servet 1, CH-1211 Geneva, Switzerland

**Keywords:** Hematopoiesis, Oxygen radicals, Amine oxidase, Transplantation, Stem cells

## Abstract

Although the development of hematopoietic stem cells (HSC) has been studied in great detail, their heterogeneity and relationships to different cell lineages remain incompletely understood. Moreover, the role of Vascular Adhesion Protein-1 in bone marrow hematopoiesis has remained unknown. Here we show that VAP-1, an adhesin and a primary amine oxidase producing hydrogen peroxide, is expressed on a subset of human HSC and bone marrow vasculature forming a hematogenic niche. Bulk and single-cell RNAseq analyses reveal that VAP-1^+^ HSC represent a transcriptionally unique small subset of differentiated and proliferating HSC, while VAP-1^−^ HSC are the most primitive HSC. VAP-1 generated hydrogen peroxide acts via the p53 signaling pathway to regulate HSC proliferation. HSC expansion and differentiation into colony-forming units are enhanced by inhibition of VAP-1. Contribution of VAP-1 to HSC proliferation was confirmed with mice deficient of VAP-1, mice expressing mutated VAP-1 and using an enzyme inhibitor. In conclusion, VAP-1 expression allows the characterization and prospective isolation of a new subset of human HSC. Since VAP-1 serves as a check point-like inhibitor in HSC differentiation, the use of VAP-1 inhibitors enables the expansion of HSC.

**Supplementary Information:**

The online version contains supplementary material available at 10.1007/s00018-021-03977-6.

## Introduction

Hematopoietic stem cells give rise to all hematopoietic cells through self-renewal and differentiation [1, 2]. Postnatally HSC reside in the bone marrow (BM) niche [3], but for therapeutic purposes they are also collected from the cord or adult blood [4, 5]. Traditionally HSC have been thought to give successively rise to multipotent and unipotent progenitors, which then produce different lineages of hematopoietic cells [6, 7]. High-resolution molecular analyses, trajectory analyses and cell-fate mapping studies have recently challenged this concept, and suggest the presence of a previously unknown heterogeneity among human hematopoietic progenitors [8–10]. The HSC and their early progenitors (Lin^−^CD34^+^CD38^−^ cells) are undifferentiated cells [11] and they gradually mature to more differentiated progeny cells (Lin^−^CD34^+^CD38^+^), which have heterogenic transcriptional signatures [12] and are lineage-restricted progenitors containing all major branches of bone marrow hematopoiesis [13]. However, it is evident that not all cell differentiation properties are reflected in the transcriptome, and thus alternative levels, possibly epigenetic, proteomic or microenvironmental, controlling cell fate do exist [14–17].

Vascular adhesion protein-1 (VAP-1) is a transmembrane protein also known as amine oxidase copper-containing 3 and semicarbazide-sensitive amine oxidase (SSAO) [18]. The extracellular amine oxidase activity of VAP-1 catalyzes oxidative deamination of amines into their corresponding aldehydes and produce ammonia and hydrogen peroxide, one of the reactive oxygen species (ROS) [19]. Interestingly, ROS regulate the function of HSC [20, 21]. In particular, high levels of ROS restrict the life-span of HSC [22, 23].

In this work, we describe the characteristics of a unique human VAP-1^+^ HSC subpopulation within undifferentiated HSC and its role in controlling HSC proliferation in vitro and in vivo. These data offer new molecular insights into the heterogeneity of HSC, define VAP-1 produced hydrogen peroxide as a check point-like inhibitor for HSC proliferation and may provide new ways for HSC expansion for clinical use in the future. Moreover, by using VAP-1 knockout mice and VAP-1 inhibitor treatments we demonstrate that VAP-1 in BM vasculature supports HSC expansion.

## Materials and methods

### Mice and human samples

Fresh human BM cells from 11 donors from AllCells, Lonza and Helsinki University Hospital and cord blood (CB) cells from Turku University Hospital were obtained. Anonymous human samples were obtained with the permission of the Ethical Committees of Turku and Helsinki University Hospitals. NBSGW mice (NOD.Cg-KitW-41 J Tyr + Prkdcscid Il2rgtm1Wjl/ThomJ) were from the Jackson Laboratory. Full VAP-1 knockout (KO) mice [24], VAP-1 knock-in (KI) mice, which express enzymatically inactive VAP-1 [25] and their wild type (WT) controls (C57BL/6 J WT mice) were littermates of heterozygous VAP-1-KO animals from our own colonies. All experimental mice were sex- and age-matched. In bone marrow transplantation studies (utilizing LJP-1586 treatment) female mice were used because of their better acceptance of transplants [26]. All other studies were performed using both male and female mice. The mice were handled in accordance to the institutional animal care policy of the University of Turku and following national and EU guidelines. All animal experiments were approved by the Finnish Animal Ethics Committee.

### Flow cytometry and cell sorting

Detailed information about the antibodies used in this study is provided in the Supplemental Table 1.

Fresh human BM and CB cells were first incubated with 5% human serum and human IgG (100 µg/ml) for 15 min at room temperature (RT) in order to reduce staining background for the fluorescein isothiocyanate (FITC) labeled anti-VAP-1 antibodies. The cells were then stained with the monoclonal antibodies 1B2, TK8-14, and/or JG-2, which recognize different epitopes of human VAP-1, or with commercial control mouse IgG and IgM against chicken bursal epithelium as isotype controls. All antibodies were conjugated to FITC using standard procedures. The following additional HSC marker antibodies were also used: APC-conjugated mouse anti-CD34, PE-Cy7-conjugated mouse anti-CD34, PE-conjugated mouse anti-CD34, PE-conjugated rat anti-CD49f, PE-Cy7-conjugated mouse anti-CD45RA, PerCP-Cy5.5-conjugated mouse anti-CD45RA, BV421-conjugated mouse anti-CD38, APC-conjugated mouse anti-CD38, BV510-conjugated rat anti-CD49f, BV605-conjugated mouse anti-CD90, APC-conjugated mouse anti-Lineage cocktail, and APC-Cy7-conjugated mouse anti-CD90. Cells were incubated with antibodies for 15 min at 4 °C. Live cells were identified based on forward and side scatter plots. Cells were sorted on a FACSAria IIu instrument (BD Biosciences) or analyzed with a LSR Fortessa instrument (BD Biosciences) using FlowJo software (Tree Star).

Mouse BM cells were collected using a BM Harvesting & Hematopoietic Stem Cell Isolation Kit (Millipore). Cells were stained with a rabbit anti-VAP-1 polyclonal antibody, a rat anti-VAP-1 monoclonal antibody 7–106 (IgG2b), negative controls (rabbit IgG or rat IgG2b), followed by appropriate FITC-conjugated goat anti-rabbit IgG and FITC-conjugated anti-rat IgG secondary antibodies. APC-conjugated anti-mouse Lineage cocktail, a PE-Cy7-conjugated rat anti-CD127 antibody, a PE-CF594-conjugated rat anti-CD135 antibody, a FITC-conjugated rat anti-CD41 antibody, a Brilliant Violet 510-conjugated rat anti-c-Kit antibody, a PE-Cy7-conjugated rat anti-CD48 antibody, a PE-conjugated rat anti-CD150 antibody, and a PE-conjugated rat anti-Sca-1 antibody were incubated for 30 min on ice. Samples were evaluated on an LSR Fortessa instrument, and data were analyzed using FlowJo software (Tree Star).

### Immunocytochemistry

FACS sorted CD34^+^ CB cells were spinned on glass slides by centrifugation at 1000 rpm for 5 min, and fixed with cold acetone for 5 min. For immunofluorescence assays, cytospined cells were blocked with 5% human AB serum. Cells were stained with either combination of FITC conjugated rabbit anti-VAP-1 polyclonal antibody, TK8-14 and JG-2 monoclonal antibodies; or combination of FITC conjugated pre-bleed serum from the same rabbit, and isotype-control mouse IgG2a. Finally, the cells were mounted using Prolong Gold anti-fade reagent with 4′,6-diamidino-2-phenylindole (DAPI; Molecular Probes). Images were acquired using an Olympus BX60 microscope. Background subtraction and adjustments of brightness and contrast were performed using ImageJ software.

Frozen murine tissue sections. (5 μm thick) were stained with a rabbit anti-VAP-1 primary antibody (made against recombinant human VAP-1 but also recognizing mouse VAP-1 [27]) followed by an Alexa Fluor 546-conjugated goat anti-rabbit IgG (H + L) secondary antibody (Invitrogen). The following directly conjugated antibodies were also used: eFluor 450-conjugated rat anti-mouse CD150 (eBioscience), APC-conjugated rat anti-mouse Lineage antibody cocktail (BD Biosciences), Alexa Fluor 488-conjugated rat anti-mouse CD31 (Biolegend). The sections were mounted using ProlongGold anti-fade reagent (Molecular Probes). Images were acquired using a LSM780 confocal microscope (Carl Zeiss) or an Olympus BX60 microscope and analyzed using Zen 2010 (Carl Zeiss) or ImageJ software.

For whole-mount staining a mouse femur was fragmented into small pieces, which were incubated with 5% normal mouse serum for 10 min at RT with gentle rotation. Thereafter, BM pieces were incubated with a rabbit anti-VAP-1 antibody (1:500) overnight at 4 °C without rotation, washed with PBS for 30 min at RT, and then incubated with Alexa Fluor 546-conjugated anti-rabbit IgG for 15 min at RT with gentle rotation. After washing with PBS for 30 min at RT with gentle rotation, BM pieces were incubated with APC-conjugated anti-CD31 and Alexa Fluor 488- conjugated anti-PV-1 (MECA-32) for 30 min at RT. Finally, BM pieces were washed with PBS for 10 min at RT with gentle rotation. Images were acquired using a 3i Marianas spinning disk confocal microscope (Intelligent Imaging Innovations, Inc) equipped with a Plan-apochromat 20 × /0.8 objective (Carl Zeiss), a Yokogawa CSU-W1 scanner, and a Hamamatsu sCMOS Orca Flash 4 (2048 × 2048 pixels) camera. Total of 57 optical sections with a slice thickness of 0.63 μm were acquired. Images were analyzed using SlideBook 6 software (Intelligent Imaging Innovations, Inc.).

### RNAseq analysis

Fresh human BM (*n* = 4, Lonza) were first incubated with 5% human serum and human IgG (100 µg/ml) for 15 min at RT. Then the BM cells were stained with the FITC conjugated monoclonal antibodies 1B2, TK8-14, and/or JG-2. The following additional HSC marker antibodies were also used: PE-Cy7-conjugated mouse anti-CD34, PE-conjugated rat anti-CD49f, PerCP-Cy5.5-conjugated mouse anti-CD45RA, APC-conjugated mouse anti-CD38, BV421-conjugated rat anti-CD90, APC-conjugated mouse anti-Lineage cocktail. Cells were incubated with antibodies for 15 min at 4 °C. Live cells were identified based on forward and side scatter plots. Cells were sorted on a Sony SH800 cell sorter with class A2 Level II biosafety cabinet using 130 µm microfluidic sorting chips.

Total of 117–150 VAP-1^+^ and VAP-1^−^ HSC from each individual were used for sequencing. Sequencing libraries were generated using: SMART-Seq v4 Ultra Low Input RNA Kit, Takara (012,516), Illumina Nextera XT DNA Library Preparation, Illumina (15,031,942), SMART-seq v4 Ultra Low Input RNA Kit (Takara), Nextera XT DNA Library Preparation Kit (Illumina), Nextera XT Index Kit V2 (Illumina). The libraries were sequenced with HiSeq 3000 (Illumina). After library preparation, quality was ensured using Agilent Bioanalyzer 2100. Sequencing was performed at the Finnish Functional Genomics Centre (FFGC) using Hiseq3000 Next-Generation Sequencing platforms (Illumina). 260–320 M reads/lane data was generated Hiseq3000 run. Raw data were delivered as a fastq format (FFGC). The RNA-seq data have been deposited to the Gene Expression Omnibus with SRA accession number PRJNA594799.

RNA-sequencing data analyses followed that of Kumar et al. [28] and included pre-processing of read data using Trimmomatic [29], gap-aware alignment of the read data to the human reference genome (Ensembl GRCh38) with the guidance of the EnsEMBL reference gene models (EnsEMBL v82) using the 2-pass per-sample mapping approach in STAR [30] and read summarization against EnsEMBL v82 gene and exon features using Rsubread [31]. Default parameters were used with the exception that reads were allowed to be assigned to more than one matched meta-feature in feature counting. Quality statistics was generated using FastQC and RNA-SeQC [32] and manually inspected.

Low expressed genes were removed from the dataset as a preliminary step. A gene was considered not expressed if the counts per million (CPM), standardized by the corresponding library size, were less than one in at least half of the samples in both VAP-1^+^ and VAP-1^−^groups. After gene filtering the dataset contained a total of 14,943 expressed genes, down from 60,619 total genes. Expression estimates were normalized with the Trimmed Mean of M-values (TMM) method [33] in all subsequent analyses.

### GSE and pathway analyses

Gene set enrichment analysis (GSEA, pre-ranked) was performed using the tool from the Broad Institute [34]. Pathway analyses were performed using STITCH database (http://stitch.embl.de/). To construct the network, interactions with a minimum confidence score 0.40 were considered significant and extracted for constructing the PPI network.

### Quantitative PCR

To detect *TPX2, CDCA8, PCNA, MLLT3* and *TYMS* FACS sorted human BM VAP-1^+^ and VAP-1^−^ HSC (Lin^−^CD34^+^CD38^−^ CD45RA^−^ CD90^+^) were used. SuperScript IV CellsDirect cDNA Synthesis Kit (ThermoFisher Scientific) was applied for direct cDNA synthesis using sorted cell lysis protocol. One cell equivalent cDNA was used for RT-PCR using TaqMan Fast Advanced Master Mix (ThermoFisher Scientific). The following TaqMan Assays were used: Hs00187842_m1 (B2M), Hs00201616_m1 (TPX2), Hs00983655_m1 (CDCA8), Hs00427214_g1 (PCNA), Hs00426586_m1(TYMS) all from ThermoFisher Scientific. cDNA was analyzed by real-time qPCR (7900HT Fast Real-Time PCR System, Applied Biosystems). The results were analyzed in Applied Biosystems qPCR analysis modules. The expression values were normalized using β2 microglobulin expression as endogenous controls.

### scRNAseq analysis

Fresh human BM cells (Lonza) from one donor were first incubated with 5% human serum for 15 min at RT. Then the BM cells were stained with the FITC conjugated monoclonal antibodies 1B2, TK8-14, and/or JG-2 and PE-Cy7-conjugated mouse anti-CD34 as well as with APC-conjugated mouse anti-Lineage cocktail. Live cells were identified based on forward and side scatter plots. Cells were sorted on a Sony SH800 cell sorter with class A2 Level II biosafety cabinet using 130 µm microfluidic sorting chips. Total of four samples, two technical replicates of each (VAP-1^+^ and VAP-1^−^ populations) were immediately loaded into the 10XGenomics Chromium controller for gel-bead-in-emulsion formation (Turku Bioscience Single-cell Omics core facility and Finnish Functional Genomics Centre supported by University of Turku, Åbo Akademi University and Biocenter Finland).

Single Cell 3ʹ Reagent Kits v3.1 (Dual Index) were used according to the manufacturer’s protocol (CG000315). In brief, for each sample, 30–40 μl of single-cell suspension at concentration of 400 cells/μl was loaded on a separate lane of the Next GEM Chip G, targeting at recovery of approximately 10,000 cells per sample. During the run, single-cell barcoded cDNA is generated in nanodroplet partitions. The droplets are subsequently reversed, and the remaining steps are performed in bulk. The barcoded cDNA was amplified using 12 PCR cycles using a Veriti thermal cycler (ThermoFisher). The amplified cDNA was then subjected to fragmentation, end repair and A-tailing, adapter ligation, and sample index PCR (13 cycles). The gene expression libraries were sequenced using an Illumina NovaSeq 6000 and an S1 flow cell with the following read length configuration: Read1 = 28, i7 = 10, i5 = 10, Read2 = 90. The raw data was processed using Cell Ranger 5.0.1. with GRCh38 as the reference genome.

### Bioinformatics of single-cell RNA sequencing

Previously sequenced HSC data (GEO accession code GSM3305359) were downloaded from NCBI [10]. Seurat (https://satijalab.org/seurat/) (version 3.1) [35] implemented in R (version 3.6) under anaconda environment was applied to reduce the dimension of HSC-derived datasets. Data generated in this manuscript were analyzed in R version 4.0.5 using Seurat suite version 4.0.1 under anaconda environment. Cells were excluded, if the number of genes detected was below 100 or the percentage of mitochondrial genes above 20% followed by log-normalization using “NormalizeData” on a scale factor as 10,000. All variable genes were detected by “FindVariableFeatures” and genes passing the “vst” filter were included in the following analysis. Linear transformation was done using “ScaleData” function. Principal component analysis (PCA) was performed using “RunPCA” function and reported by “JackStraw” procedure and only significant PCs were selected using the elbow method to perform dimension reduction and clustering. Cells were projected in 2D space using non-linear dimensional reduction techniques (UMAP) with default parameters. For differential expression analysis to find DEGs among different clusters, “FindMarkers” function with default parameters was applied. Marker genes of different clusters were selected using Wilcoxon Rank Sum test. Signature gene expression was calculated as percentage of sum counts of selected genes vs all expressed genes. The percentage ratio was added as a feature value to each cell and multiplied by 100. Sub-clustering analysis was performed according to a recommended procedure by Seurat. Trajectory was constructed with the Monocle 2 package (v2.10.1) [36] according to the documentation (http://coletrapnell-lab.github.io/monocle-release) and kernel density estimation was done using Nebulosa R package (https://github.com/powellgenomicslab/Nebulosa).

### Meta and Gene Ontology (GO) analyses

GO biological process and pathway enrichment analyses were performed using Metascape (https://metascape.org/) [37]. From bulk RNAseq, 687 VAP-1^+^ and 378 VAP-1^−^ genes (fold change > 1, *p*-value < 0.05); and from scRNAseq 371 VAP-1^+^ and 50 VAP-1^−^ genes (according to Seurat recommended way) were applied for analysis. G1/S and G2/M cell cycle genes and aorta-gonad-mesonephros containing HSPC1, HSPC2 and HSPC3 gene signatures were collected from previously defined gene sets [38, 39].

### Isolation of CD34^+^ cells and sorting of VAP-1^+^and VAP-1^−^ HSC from human umbilical CB

CD34^+^ cells from human umbilical CB were isolated via a two-step procedure using Ficoll-Plaque gradient centrifugation (Amersham Pharmacia Biotech, Uppsala, Sweden) and EasySep Human Cord Blood CD34 Positive Selection Kit II (STEMCELL Technologies). For batch and single cell sorting of VAP-1^+^ and VAP-1^−^ cells from CB we used a Sony SH800 cell sorter. This sorter applies low shear stress on cells allowing better survival during cell culture. CD34^+^ cells separated by magnetic beads (Stem Cell Technologies) were sorted into VAP-1^+^ and VAP-1^−^ HSC using the following markers: Lineage^−^CD34^+^CD38^−^CD90^+^CD45RA^−^CD49f^+^. The sorted cells were then cultured as single cells in 96 well plates with Methocult GF + H4435 medium (Stem Cell Technologies). Single cells were cultured for 14 days, washed and replated as single colonies in 24 well plates for another 12 -14 days. Colonies were evaluated for CFUs by light microscopy.

### Liquid cultures, colony-forming unit (CFU) and long-term culture-initiating cell (LTC-IC) assays

For human umbilical CB cells, an antibody-based EasySep kit was used to enrich CD34^+^ CB cells, which were subsequently stained with anti-CD38 and anti-CD34 antibodies. CD38^−^CD34^+^ cells were sorted using a FACSAria IIu instrument (BD Biosciences) and then cultured in StemSpan SFEM medium II (STEMCELL Technologies) containing human stem cell factor (100 ng/ml), FMS-like tyrosine kinase 3 ligand (100 ng/ml), and thrombopoietin (50 ng/ml) (all from Peprotech). Cells were seeded at a density of 1 × 10^3^ per ml. LJP-1586 was added immediately after plating when indicated. Cultures were maintained for 21 days, and half of the medium was replaced by new medium containing the same cytokines and LJP-1586 on days 5, 8, 12, 15, and 18.

Five hundred cryopreserved and thawed human BM CD34^+^ cells (AllCells) were cultured in complete methylcellulose-based medium (H4436, STEMCELL Technologies) with or without LJP-1586. After 12 days, cells were resuspended, re-plated the second time after increasing the volume of the culture by tenfold, resuspended again, and re-plated the third time after increasing the volume of the culture by fivefold. The total number of colonies was counted at 14 days after plating.

The mouse colony-forming unit (CFU) assay was performed using methylcellulose-based MethoCult GF M3434 medium (STEMCELL Technologies). BM and peripheral blood cells were seeded in triplicate, and colonies were scored after 12 days. The mouse long-term culture-initiating cell (LTC-IC) assay was performed according to the manufacturer’s instructions.. Briefly, stromal feeder layers were prepared using total BM cells isolated from the femurs and tibias of WT and VAP-1-KO mice. BM was cultured in long-term culture medium (MyeloCult M5300 containing 10^−6^ M hydrocortisone; STEMCELL Technologies) for 2 weeks, during which time half the medium was replaced every week. Feeder layers at 80% confluency were irradiated with 1500 cGy using an X-ray source. Resuspended and unprocessed BM cells from WT and VAP-1-KO mice were cultured in long-term culture medium on the irradiated feeder layer. Cultures were maintained for 4 weeks, during which time half the medium was replaced every week. Non-adherent cells were harvested and grown in methylcellulose-based medium (MethoCult GF M3434, STEMCELL Technologies) for an additional 12 days. Plates were scored as positive or negative to yield the LTI-IC frequency responding or nonresponding.

### Measurements of ROS production

Human CD34^+^ BM cells were liquid cultured for nine days in StemSpan SFEM medium II (STEMCELL Technologies) containing human stem cell factor (100 ng/ml), FMS-like tyrosine kinase 3 ligand (100 ng/ml), and thrombopoietin (50 ng/ml) (all from Peprotech) with or without LJP-1586. After nine days, the cells were stained with anti-CD38 and anti-CD34 antibodies, washed using DMEM, centrifuged and resuspended in 100 µl DMEM. ROS were detected by DHR-123 reagent (Molecular Probes). For this, DHR-123 was diluted in DMSO and kept as a 5 mM stock solution at  – 20 °C for single use. The aliquots were thawed, diluted 160 times (30 µM) just before adding 12.5 µl to the HSC suspended in 100 µl DMEM to a final concentration of 3 µM. The cells were then incubated for 10 min at 37 °C and followed by activation with Phorbol 12-myristate 13-acetate (PMA) (Sigma-Aldrich. The stock solution of PMA was frozen at 1 mg/ml in DMSO, freshly thawed and diluted 500 times in order to add 12.5 µl to a final concentration of 200 ng/ml. After 20 min at 37 °C, the cells were washed with PBS, resuspended and analyzed by flow cytometry. The red DHR123 turns to green after oxidation. CD38^−^ and CD34^+^ positive cells were gated and fluorescence intensity of oxidized DHR-123 was measured from the filter channel 530 nm/30 nm using LSR Fortessa instrument (BD Biosciences) and analyzed by FlowJo software (Tree Star).

### Bone marrow transplantations

Human fresh frozen BM CD34^+^ cells (LONZA) were thawed and stained with APC conjugated mouse anti-Lineage cocktail, PE-Cy7-conjugated anti-CD34 and FITC–conjugated monoclonal antibodies 1B2, TK8-14, and JG-2 against different epitopes of human VAP-1. For batch cell sorting of VAP-1^+/lo^ and VAP-1^−^ cells we used a Sony SH800 cell sorter. The NBSGW (immune-deficient, c-Kit-deficient) mice not needing irradiation to accept human cells were used as BM  recipients. In the VAP-1^−^ group 19,000 cells and in the VAP-1^−^ + VAP-1^+/lo^ group 16,250 VAP-1^−^ cells and 2750 VAP-1^+/lo^ cells were intravenously injected per animal.

For inhibitor studies, human mononuclear umbilical CB cells were collected using Ficoll-Plaque gradient centrifugation (Amersham Pharmacia Biotech, Uppsala, Sweden). Two million mononuclear CB cells were intravenously injected per animal. One day after the transplantation mice were intraperitoneally injected with VAP-1 inhibitor, LJP-1586 [40] at a dose of 10 mg/kg or with 100 µl of PBS as a control three times for BM cells and two times for CB cells in a week for total of six weeks. At the end of the treatment the mice were sacrificed and BM were collected. BM cells were stained for APC conjugated anti-mouse CD45, BV421 conjugated anti-human CD45, APC-Cy7 conjugated anti-mouse CD45, PE conjugated anti-human CD45, PE-Cy7-conjugated mouse anti-CD34 and APC-conjugated mouse anti-CD38. Samples were run on LSR fortessa and the data was analyzed with FlowJo.

### Irradiation

A week before irradiation the WT and KO mice were put into individually ventilated cages, and provided with acidified water (pH = 2.8). The mice were given a single dose of 950 cGy at a rate of 200 cGy/minute.

### LJP-1586 treatment

Cohorts of ten 2-week-old C57BL/6 J mice were intraperitoneally injected with LJP-1586 at a dose of 10 mg/kg (chosen based on the previous publications [40–42]) or with 100 µl of PBS as a control for 1 week or 6 weeks.

### Statistical analyses

All results are displayed as mean ± SEM. Two-tailed, unpaired Student’s t-test was used and *p*-value < 0.05 was used as a threshold for rejecting the null hypothesis. All statistical analyses of RNAseq data were performed with R 3.6.1. (R Foundation for Statistical Computing, Vienna, Austria. URL https://www.R-project.org/). Model estimates were adjusted for the paired experimental design by introducing a donor effect parameter in the linear model. Let ${y}_{gij}$ be the observed value of gene $g$ corresponding to donor $i$ in group $j$, ${\mu }_{gi}$ the average effect of individual $i$ in gene $g$, ${\theta }_{gj}$ the average effect within group $j$. All employed statistical models defined the expected value to be equal to

$$E[y_{gij} ] = \mu_{gi} + \theta_{gj}$$

Heatmaps, multidimensional scaling, gene enrichment analysis, and pathway analysis all used log2-counts per million (logCPM) values normalized with the *voom* algorithm from the *limma* package [43]. Differential expression analysis was instead done with package *edgeR* [44] using its quasi-likelihood (QL) pipeline [45]. The linear model is in this case extended by assuming the CPM values to follow a Negative Binomial distribution with a variance equal to

$$V[y_{gij} ] = \sigma_{g}^{2} E[y_{gij} ]\left( {1 + \phi E[y_{gij} ]} \right)$$

Parameter ${\sigma }_{g}$ is the standard deviation associated with gene $g$ while $\phi$ represents the overall biological variability across all genes, estimated from the data prior the expression analysis. Computed p-values were adjusted for multiple hypothesis testing with the *fdrtool* package [46].

## Results

### VAP-1 is expressed on HSC in human BM and cord blood

**Fig. 1:**
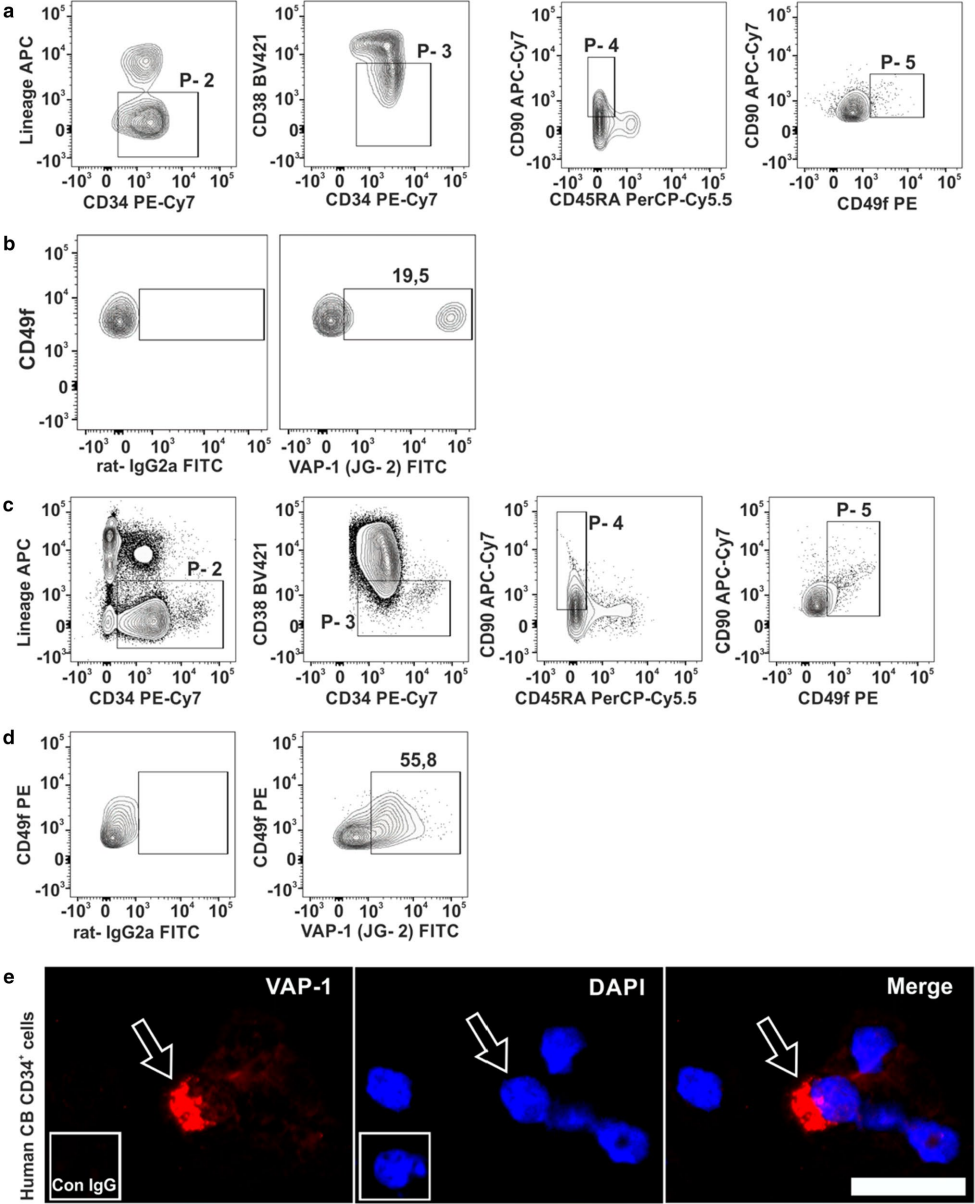
VAP-1 expression on human BM and CB HSC. **a** Flow cytometric identification of HSC in human BM. The plots show the sequential gating strategy for identifying Lineage^−^CD34^+^CD38^−^CD90^+^CD45RA^−^CD49f^+^ HSC (gate P-5). **b** Staining of gate P-5 HSC using the anti-VAP-1 antibody JG-2 or an isotype-matched negative control antibody. **c** Identification of human CB HSC from FACS sorted CD34^+^ CB cells. **d** CB HSC (gate P-5) analyzed for VAP-1 expression as in  **b**. **e** Expression of VAP-1 on FACS-sorted CD34^+^ CB cells using immunofluorescence staining of cytospin slides. The arrow points to a VAP-1^+^ cell. The inset shows a negative control staining. Scale bar 50 µm. In **b** and **d**, the numbers indicate the percentages of VAP-1^+^ cells (BM 19.5 ± 2%, *n* = 5; CB 51.8 ± 8.7%, *n* = 10

AOC3 gene coding for VAP-1 was almost undetectable in HSC in database searches [47] (http://satijalab.org/cd34/, fig. S1a). Since also many prototype HSC protein markers are transcribed at a notably low level in HSC, we also stained for VAP-1 in bone marrow HSC using flow cytometry. Analysis of Lineage^−^CD34^+^CD38^−^ cells, which were also CD90^+^CD45RA^−^CD49f^+^, revealed that a subset of these HSC (19.5 ± 2%, *n* = 5) expressed VAP-1 on the cell surface (Fig. 1a, b).

CD34^+^ cells isolated from human umbilical CB and analyzed using the same HSC markers also expressed VAP-1 (Fig. 1c, d). This finding was confirmed using two other VAP-1-specific monoclonal antibodies (1B2 and TK8-14) which recognize different epitopes of VAP-1 (fig. S1b and c). We also confirmed VAP-1 expression in HSC by cytological stainings of FACS-sorted CD34^+^ cells from CB. In line with the flow cytometric data, we observed that only few cells had high level of VAP-1 protein expression, while the rest were VAP-1 negative (Fig. 1e). In conclusion, although practically absent at mRNA level, VAP-1 protein is present on the cell surface in a small subset of HSC in BM and CB.

### VAP-1^+^ HSC are undifferentiated HSC

**Fig. 2:**
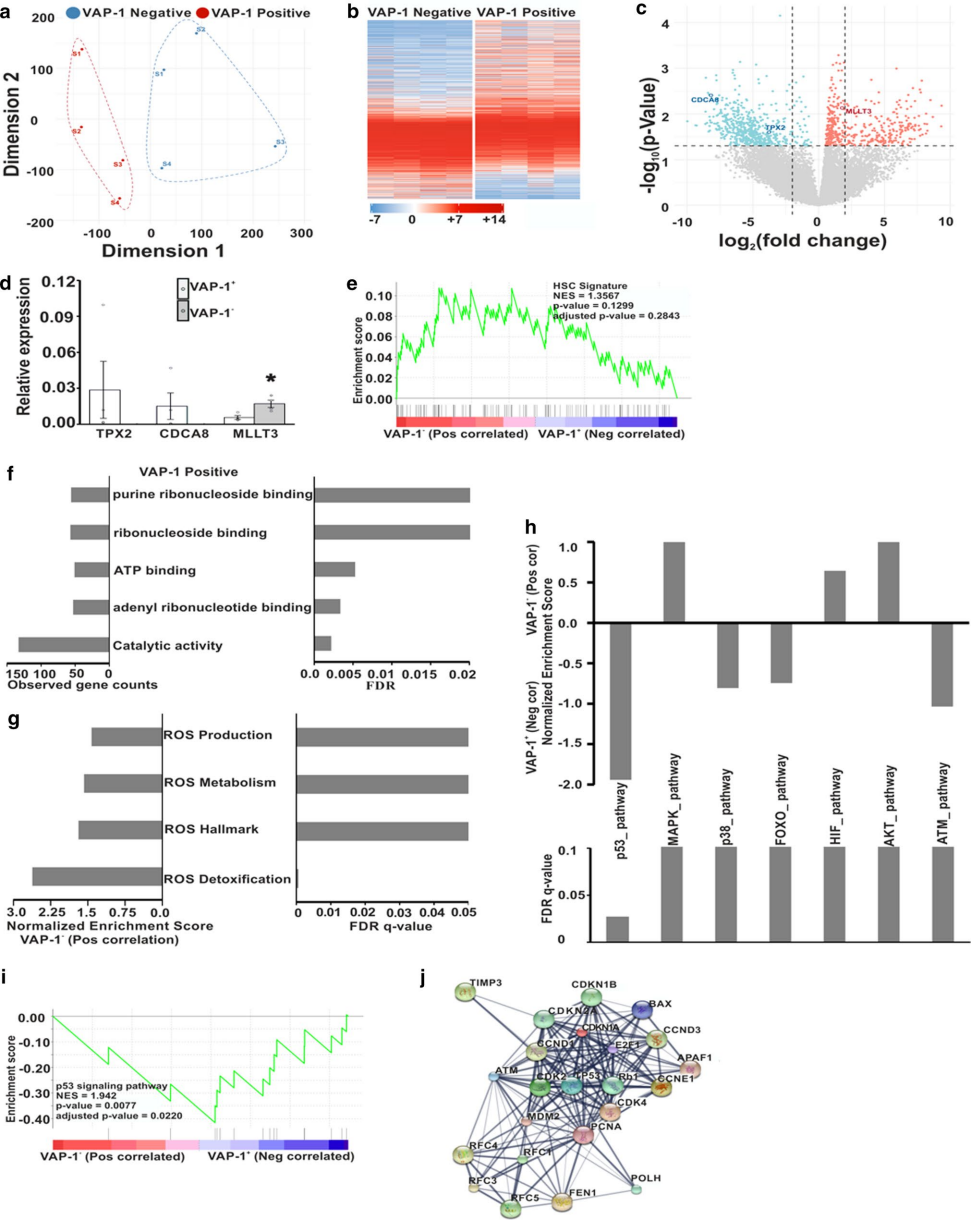
VAP-1^−^ and VAP-1^+^ HSC from adult human BM are transcriptionally different. **a** Population-distance analysis of VAP-1^−^ and VAP-1^+^ HSC (Lin^−^CD34 + CD38^−^ CD45RA^−^ CD90^+^CD49f^+^ cells) presented as a multidimensional scaling plot of the bulk RNAseq data in two dimensions. Each point represents a single biological replicate. **b** A heatmap of genes differentially expressed in VAP-1^−^ and VAP-1^+^ HSC. Colors represent the relative expression of a given gene in comparison with the median of all samples. The heat map color scheme with the corresponding log2-values is given, *n* = 4 in both groups. **c** A volcano plot showing the distribution of gene expression changes in VAP-1^−^ and VAP-1^+^ HSC. Genes were plotted as − log10(*p*-value) on the y axis against the log2(fold-change) on the x axis. **d** Quantitative real-time polymerase chain reaction (qPCR) of selected differentially expressed genes in VAP-1^+^ and VAP-1^−^HSC. The expression intensity was normalized with the expression level of *β2 microglobulin (*B2M). Two samples independent from those used in RNAseq were analyzed (*p*-value < 0.05). **e** Gene set enrichment analysis (GSEA) of the HSC signature genes in VAP-1^−^ and VAP-1^+^ HSC. Normalized enrichment score (NES). **f** Gene ontology (GO) molecular signaling pathway analysis for DEGs. Shown are the top five pathways (FDR < 0.02 and > 50 observed genes. **g** GSEA for ROS production, hallmark, metabolism and detoxification. NES and FDR-q values are shown. **h** GSEA for p53, MAPK, p38, FOXO, HIF, AKT and ATM signaling pathways. NES and FDR-q values are shown. **i** The enrichment plot of the p53 signaling pathway. **j** A protein–protein interaction (gray lines, the thickness of which represents confidence and strength of the association) network of the enriched genes of the p53 signaling pathway in VAP-1^+^ HSC. Proteins are shown as spheres

Next we checked, whether there are differences in transcriptomics between BM VAP-1^+^ and ^–^ HSC. RNAseq analyses of FACS-sorted human BM VAP-1^+^ and ^–^ HSC populations (Lin^−^CD34^+^CD38^−^CD45RA^−^ CD90^+^CD49f^+^) revealed marked differences in their transcriptomics (Fig. 2a and Table S2). Among 14,000 expressed genes 1387 genes, including cytokines, G-protein coupled receptors, transmembrane receptors and kinases (Table S2), had more than fivefold log change in expression between the VAP-1^+^ and VAP-1^−^ HSC populations. Of all differentially expressed genes (Fold change > 2, *p*-value < 0.05), 516 were up-regulated in the VAP-1^+^ and 183 in the VAP-1^−^ populations (Fig. 2b, c) and Table S2, 3). Certain unique DEG were further tested using real-time qRT-PCR to confirm the differential expression on VAP-1^+^ and VAP-1^−^ HSC (Fig. 2d). In line with the bulk RNAseq data, *TPX2* and *CDCA8* were found in VAP-1^+^ cells but were almost undetectable in VAP-1^−^ cells, whereas *MLLT3* had more than threefold higher expression in VAP-1^−^ cells. Gene Set Enrichment Analysis (GSEA) of the human HSC associated genes, collected from molecular signatures database (EPPERT_HSC_R)[48], revealed a random distribution of HSC genes in VAP-1^+^ and VAP-1^−^ populations (Fig. 2e and Table S4). Analyses of other data sets (CB HSC signature, CD49f^+^ HSC signature) gave comparable results (Fig. S2a–n). These results indicate that both VAP-1^+^ and VAP-1^−^ populations are true HSC with distinct transcriptomes.

The top molecular pathway (with more than 50 genes involved and false discovery rate (FDR) < 0.02) in VAP-1^+^ population was catalytic activity (Fig. 2f), consistent with the ROS-producing enzymatic activity of VAP-1. VAP-1^+^ and VAP^−^ populations had comparable levels of mRNA of genes regulating the production of ROS in general (Fig. S2a–n). However, GSEA showed that ROS detoxifying genes such as superoxide dismutase 1 and 2 (catalyzing superoxide detoxification into H2O2) had significant positive correlation to VAP-1^−^ population (*p* = 0.001), suggesting that VAP-1^+^ cells regulate the level of H2O2 involved in HSC maintenance (Fig. 2g and Fig. S2e and Table S4). To check whether superoxide dismutase 1 (SOD1) protein was downregulated in VAP-1^+^ cells, we stained and analyzed cord blood cells using Lin^−^CD34^+^CD38^−^CD90^+^VAP-1^+^SOD1^+^. To check whether SOD1 protein was downregulated in VAP-1^+^ cells, we stained cord blood cells (Lin^−^CD34^+^CD38^−^CD90^+^) for VAP-1 and SOD1. We found that SOD1 protein was detected mainly in VAP-1 negative cells (*p*-value < 0.05) (Fig. S2o–p). We further analyzed p53, FOXO, p38MAPK, ATM and HIF pathways (Fig. 2h and Fig. S2a–n and Table S4), which are known to be important for ROS-dependent HSC functions. The p53 signaling pathway was highly enriched among VAP-1^+^ cells (Fig. 2i), whereas no statistically significant differences were found in p38MAPK, FOXO, ATM and HIF signaling pathways (Fig. S2a–n and Table S4). Using STITCH data base (http://stitch.embl.de/) [49] a functional association network was drawn using the core enriched genes in VAP-1^+^ population from the p53 pathway (Fig. 2j). In the top differentially expressed gene list many of the cell cycle related genes were enriched in VAP-1^+^ cells. Therefore, we used the Meta-analysis workflow in Metascape to combine DEGs with cell cycle transition genes (G1/S and G2/M) [38] to identify possible correlations. From G1/S and G2/M published gene sets encompassing 43 genes and 55 genes respectively 18% G1/S and 38% G2/M genes were enriched among VAP-1^+^ cells (Fig. S3a). During the hematopoietic stem cell generation three hematopoietic stem progenitor cell (HSPC) clusters HSPC1, HSPC2 and HSPC3 have been identified [39]. HSPC1 appears first and carries highest stemness property, HSPC2 is largely a cycling cell population, whereas HSPC3 is closest to lineage primed cells. This meta-analysis confirms that VAP-1^+^ cells have similarities with HSPC1 and HSPC2 but have no similarities with HSPC3 (Fig. S3b). Collectively these findings indicate that VAP-1^+^ HSC in BM are a functionally distinct subset with a potential to regulate peri-cellular ROS level.

### Single cell RNA-seq profiling of human BM HSC reveals two subtypes

**Fig. 3:**
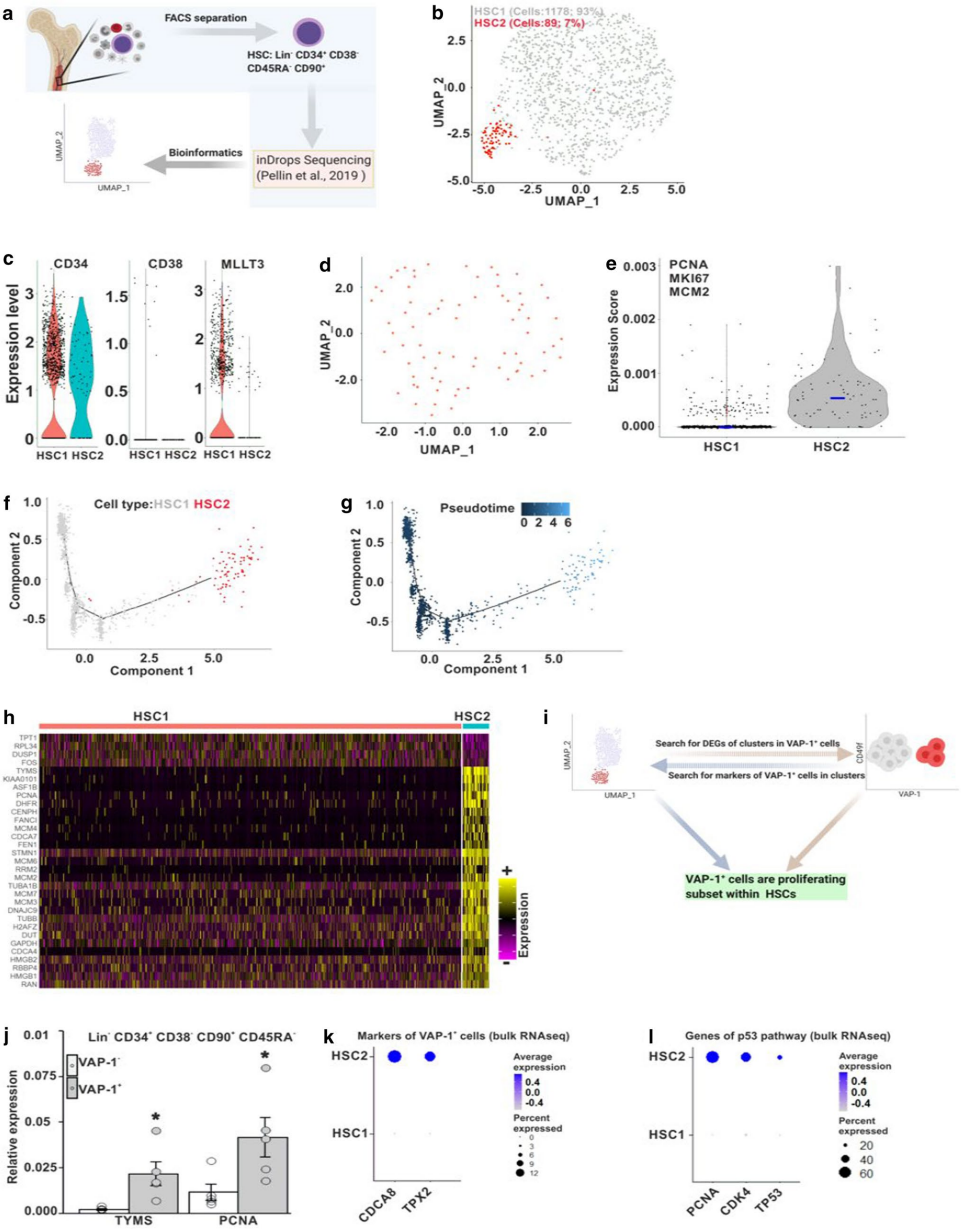
Identification of a new subclass of human BM HSC. **a** A schematic presentation of the protocol used for HSC sub-cluster identification. **b** Visualization of two major classes of HSC (HSC1 and HSC2). Each dot is a single cell colored by cluster assignment. **c** Expression of known HSC markers in HSC1 and HSC2. **d** Sub-clustering analysis of the HSC2 subset. **e** Expression of the indicated cell proliferation signature genes in HSC1 and HSC2. Horizontal bars indicate the median gene expression. **f** Single cell trajectory analysis of the differentiation path within HSC. Cells are colored according to the cluster. **g** Pseudotime trajectory analyses of HSC differentiation. The pseudotime indicator is shown at the top. **h** A heatmap showing the scaled expression of top differentially expressed genes (DEGs) in HSC1 and HSC2 clusters. The color scheme is based on z-score distribution from low (purple) to high (yellow). The left side lists the genes specific for the respective HSC subsets. **i** The outline of the protocol used for identifying proliferating VAP-1^+^ cells in the human BM HSC scRNAseq dataset. **j** qPCR validation of HSC2 genes (from a) in VAP-1^+^ HSC population (*p*-value < 0.05). **k** and **l** Dot plots showing the scaled expression levels of top genes (k) and genes of p53 pathways (l) differentially expressed in VAP-1^+^ HSC (identified in bulk RNAseq data, Fig. 2b) in HSC1 and HSC2 cells (based on the scRNAseq data). The color intensity reflects the expression level, and the circle size reflects the proportion of the cells expressing the given gene

Our FACS and RNAseq analyses suggested that VAP-1^+^ cells represent a distinct population of HSC. To resolve the heterogeneity of BM HSC (Lin^−^CD34^+^CD38^−^CD90^+^CD45RA^−^) we utilized single cell RNAseq data from Pellin et al. [10] (Fig. 3a). After the quality controls we retrieved total of 1267 high quality HSC for downstream analyses. Unsupervised clustering using Seurat pipeline identified two major cell clusters (Fig. 3b) [35]. Since both populations expressed previously described markers of HSC and, more specifically those of primitive HSC including CD34, CD38 and MLLT3 [6, 7, 50] (Fig. 3c), we tentatively named them as HSC1 (93% of cells) and HSC2 (7% of cells). In further sub-clustering with higher resolution, all 89 cells of HSC2 were randomly distributed in two dimensional graphs (Fig. 3d). Neither HSC1 or HSC2 expressed endothelial, human hemogenic endothelial [39] nor stromal or mature blood cell markers (Fig. S4a–c). Similarly, both HSC clusters were negative for leukocyte lineage markers, *CD7* and *CD19* (Fig. S4d) and apoptosis-related genes *BCL2, BCL21* and *CASP3* (Fig. S4e). Notably, both HSC1 and HSC2 were also practically negative for *AOC3* mRNA (Fig. S4f).

When comparing the two HSC clusters, common cell proliferation markers (*PCNA, MKI67, MCM2*) were characteristically highly expressed in HSC2, but not in HSC1 cells (Fig. 3e). Single cell trajectory analyses of HSC differentiation identified HSC2 as a distinct cluster, and pseudotime trajectory analysis suggested that HSC2 evolved later than HSC1 (Fig. 3f, g). Trajectories for HSC1 and HSC2 populations were confirmed using a different algorithm, monocle (http://cole-trapnell-lab.github.io/monocle-release/). These data suggest that HSC2 has features of proliferating cells on their way from dormancy towards expansion.

It is well established that many protein markers of human HSC (surface proteins, signaling molecules, transcription factors) are expressed at low or undetectable level in mRNA-based analyses. To validate the identification of the HSC1 and HSC2 clusters, we further analyzed expression of SPI1, a transcription factor important for determining the lifetime of HSC [51]. A total of 22 SPI1 cells were identified and they distributed sporadically among HSC1 and HSC2 in the two dimensional plot (Fig. S5a, b). Prototype HSC cell surface markers (*CD90, CD49f, CD33, PROM1, PTPRC*) and HSC related transcription factors (*GATA1, GATA3, HOXB5*) [6, 7, 11, 51, 52] were expressed at similar, and usually very low, levels in HSC1, HSC2 and SPI1 populations (Fig. S5c, d). Using the putative HSC2 markers *PCNA*, *MCM2* and *MKI67* identified above, we verified that HSC2 cluster is distinct from HSC1 and SPI1 clusters. (Fig. S5e). Collectively, these scRNAseq analyses support the concept that HSC2 is a unique HSC cluster.

### VAP-1^+^ HSC are the more differentiated subset within HSC

To find out, whether VAP-1 expression discriminates HSC1 and HSC2 populations we sorted VAP-1^+^ and VAP-1^−^ cells from human BM HSC (Lin^−^CD34^+^CD38^−^ CD45RA^−^ CD90^+^). Using direct qPCR from a single cell equivalent of cDNA we tested the expression of *TYMS* and *PCNA* (representative top DEGs for HSC2, see Fig. 3h). *TYMS* expression was tenfold higher (*n* = 5, *p* < 0.01) and *PCNA* expression 3.5 fold higher (*n* = 5, *p* < 0.03) in VAP-1^+^ than in VAP-1^−^ cells (Fig. 3i, j). Moreover, the bulk RNAseq VAP-1^+^ HSC markers *TPX2* and *CDCA8* (Fig. 2c), which were confirmed to be undetectable in VAP-1^−^ cells in qPCR analyses (Fig. 2d), were highly expressed in HSC2, but were practically absent in HSC1 when analyzed from the HSC scRNAseq data (Fig. 3k). In addition, major genes of p53 pathway were abundantly expressed in HSC2 (Fig. 3l), in line with the observation that p53 was the most significantly enriched pathway in VAP-1^+^ HSC in bulk RNAseq analyses (Fig. 2i). To confirm similarities of VAP-1^+^ and HSC2 populations, differentially expressed genes from bulk and scRNAseq were analyzed for Gene Ontology and pathways. Both cell populations synchronously presented multiple pathways involved in entry to cell cycle and proliferation (Fig. S6a, b). Collectively, scRNAseq data suggest that VAP-1^+^ HSC correspond to PCNA^high^ differentiated HSC2 subset, while VAP-1^−^ HSC represent the predominant HSC1 population. Thus, VAP-1 protein expression may serve as a new HSC2 subset specific surface marker.

To validate our in silico analyses, we performed scRNAseq of adult human BM VAP-1^+^ and ^−^ HSC. ScRNAseq of FACS-sorted human BM VAP-1^+^ and ^–^ HSC populations (Lin-CD34 +) revealed that proliferating HSC are mainly VAP-1^+^ HSC. Unsupervised clustering using Seurat pipeline identified six major cell clusters (Fig. S7a). VAP-1^+^ HSC contained higher proportions of Cluster 2 and Cluster 3 and lower proportions of Cluster 0 (Fig. S7b). All four samples together pointed out that VAP-1^+^ cells are substantially distinctly positioned from the VAP-1^−^ cells (Fig. S7c). Applying nebulosa to p53 pathway (Fig. S7d), bulk RNAseq VAP-1^+^ HSC markers (Fig. S7e), and HSC2 cell proliferation markers from Pellin’s data (Fig. S7f), we observed a clear expression signal in VAP-1^+^ clusters. Cell cycle gene scoring further confirmed that S.Score and G2M.Score are highly enriched among VAP-1^+^ cells (Fig. S7g). Monocle pseudo-time trajectory analysis confirmed that VAP-1^+^ evolved later than VAP-1^−^ cells (Fig. S7h and i).

### VAP-1 inhibition enhances expansion of HSC in vitro

**Fig. 4:**
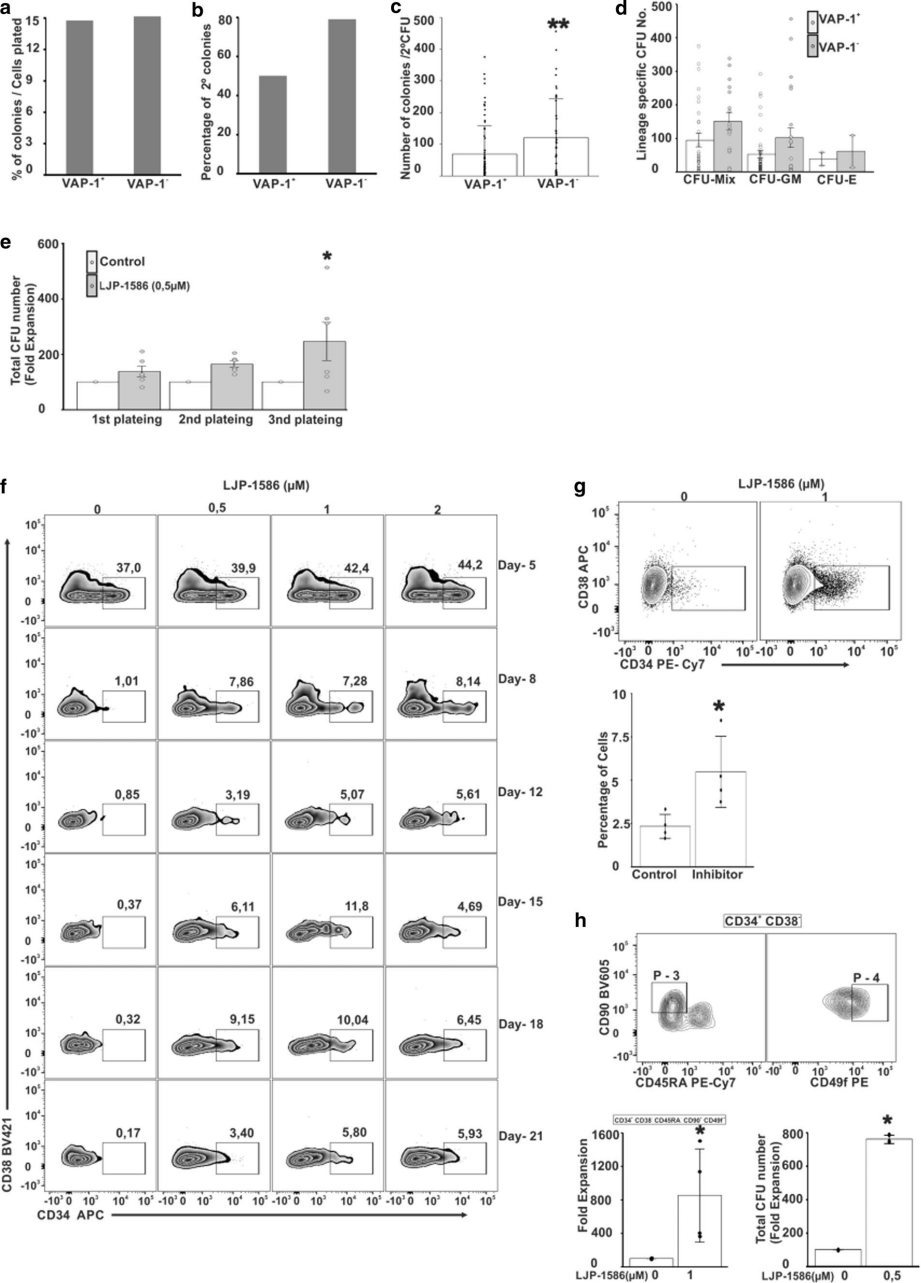
Inhibition of VAP-1 expands BM and CB HSC in in vitro cultures. **a** The number of colonies arising from single-cell sorted VAP-1^+^ and VAP-1^−^ CB. **b** The number of colonies from (**a**) re-plated and cultured for another 12–14 days. **c** The number of colonies from (**b**) counted by microscopy (*p*-value < 0.01). **d** Characteristics and number of CFU-E, CFU-GM or CFU-MIX colonies. **a**–**d**; CB collected from 60 donors. **e** Number of BM HSC in CFU assays in presence or absence of VAP-1 inhibition. Five hundred human BM-derived CD34^+^ cells were cultured under CFU conditions in presence of LJP-1586 (0.5 µM) or vehicle. *n* = two donors, triplicate cultures (*p*-value < 0.05). **f** Kinetic analyses of sorted CD38^−^CD34^+^ CB-derived cells cultured with different concentrations of LJP-1586 for 21 days. Data from one representative donor out of 3 are shown. **g** Effect of LJP-1586 (1 µM) on FACS-sorted CD38^−^CD34^+^ CB cells cultured in vitro for 15 d, *n* = 4 donors (*p*-value < 0.05). **h** Analyses of CD34 + CD38-CD45RA^−^CD90^+^CD49f^+^ HSC expansion subsequent to 15d LJP-1586 treatment measured as fold expansion and CFUs (*n* = 4) (*p*-value < 0.05)

To test the function of VAP-1 in human HSC, we performed CFU assays. Initially we sorted single HSC from CB and cultured them in vitro in a complete methylcellulose medium for 14 days (Fig. 4a). After re-plating and an additional 12–14 d incubation, 79% of plated VAP-1^−^ colonies and 50% of the plated VAP-1^+^ colonies grew (Fig. 4b). VAP-1^−^ HSC also generated substantially higher numbers of colonies (mean 121) compared to VAP-1^+^ cells (mean 69 colonies) (*p* < 0.01; Fig. 4c). Microscopically CFU-E, CFU-GM or CFU-Mix cells were identified in the progeny of both VAP-1^+^ and VAP-1^−^ cells, and the numbers of all three CFU subpopulations were increased in the cultures starting with sorted VAP-1^−^ cells, although this increase was not statistically significant, when the cell types were analyzed separately (Fig. 4d).

When BM-derived CD34^+^ cells were cultured in methylcellulose-based medium in the presence of a specific VAP-1 enzyme inhibitor LJP-1586, the number of CFUs formed was 33% higher after the first plating than the number of CFUs formed in control cultures. To determine whether these colonies contained HSC, we dissociated them into single-cell suspensions and re-plated the cells. After repeating this procedure twice (3rd plateing), the number of CFUs formed by LJP-1586-treated cultures was 246% higher than the number of CFUs formed by control cultures (*p* < 0.05; Fig. 4e). We also cultured CD34^+^ cells sorted from human CB for 21 days in StemSpan SFEM medium II containing different concentrations of LJP-1586. In control cultures the frequency of HSC decreased from 37 to 1% already in 8 days. In contrast, the frequency of HSC readily expanded in LJP-1586-treated cultures for up to 21 days (Fig. 4g). HSC expanded more than 31 times in cultures treated with 1 µM LJP -1586 and grown for 18 days compared to the control cells (not containing LJP-1586). Expansion of HSC was less efficient in cultures treated with higher or lower concentrations of LJP-1586. The degree of HSC expansion was donor-dependent, but was consistent in samples sorted from a single donor (Fig. 4f, g). On average, in LJP-1586 treated cultures CD34^+^ cells expanded 234% when compared to cultures without the inhibitor (5.4 vs. 2.3, *p* = 0.027).

Primitive HSC were further assessed using the additional markers CD45RA^−^CD90^+^CD49f^+^ (Fig. 4h). More than 12% of HSC in gate P-3 were primitive HSC (CD34^+^CD38^−^CD45RA^−^CD90^+^CD49f^+^) and the number of these was 11 times higher in LJP-1586-treated compared to non-treated cultures (Fig. 4h). Using dihydrorhodamine (DHR 123) and flow cytometry we found that ROS were reduced by 62% (MFI) when the cells were cultured with the LJP-1586 inhibitor compared to the control cells (shown for BM derived HSC in Fig. S8). In conclusion, VAP-1 inhibition in liquid cultures expands HSC (CD34^+^CD38^−^) and primitive HSC (CD34^+^CD38^−^CD45RA^−^CD90^+^CD49f^+^) compared to the untreated cells suggesting that VAP-1 may serve as a check-point inhibitor restricting HSC proliferation.

### VAP-1 inhibitors improve HCS engraftment in vivo

**Fig. 5:**
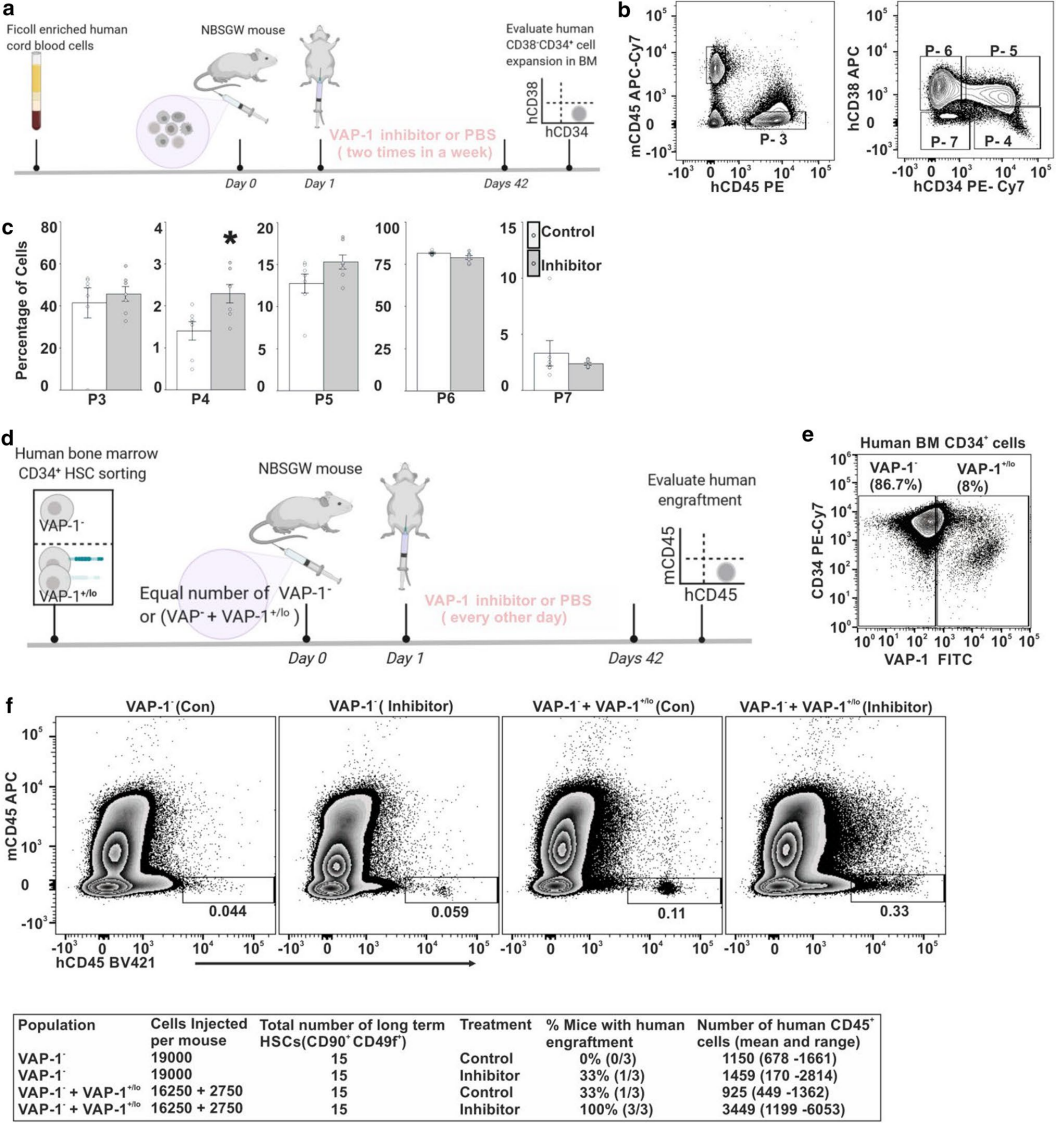
Inhibition of VAP-1 increases the engraftment potential of CB and BM HSC in NBSGW mice. **a** The experimental set up for in vivo analysis of mononuclear CB cells with VAP-1 inhibitor. **b** Gating strategy of harvested BM cells from recipients at the end of the experiment by flow cytometry using antibodies against mouse CD45, human CD45, human CD34 and human CD38. **c** Percentages of cells isolated from BM of transplanted mice (*n* = 7) within the different gates defined in (**b**) (*p*-value < 0.05). **d** The experimental scheme for in vivo analyses of VAP-1 ^–^ and VAP-1^+/lo^ HSC in the presence of VAP-1 inhibitor. **e** Batch sorting and the frequencies of VAP-1^−^ and VAP-1^+/lo^ HSC from human BM in the CD34^+^ gate. **f** In vivo engraftment of VAP-1^−^ and VAP-1^+/lo^ human BM cells in non-irradiated NBSGW mice after 42 days. Representative flow cytometric plots (percentages of engrafted cells) and quantification from each group (*n* = 3 for each group). The cut-off value for engraftment was set as 0.1

Since VAP-1 inhibitor expands HSC in vitro, we wanted to study if VAP-1 is involved in expanding HSC also in vivo. It is known that in vitro cultured HSC are not comparable with freshly isolated HSC. In vitro cultured HSC remarkably change their properties within 5 days [53], whereas HSC are maintained in vivo without any visible exhaustion [54]. In addition, HSC can divide asymmetrically in vitro but not all proteins segregate, and asymmetric segregation is clearly defined only until granddaughter cells [55]. Thus, long term in vitro cultured HSC cannot be used in in vivo transfer studies (and this was confirmed by our initial trial; data not shown). Therefore, for an in vivo experimental setting we used freshly isolated human HSC and transplanted them to NBSGW mice, which were treated by intraperitoneal administration of VAP-1 inhibitor. We first transferred CB derived mononuclear cells (including HSC) to NBSGW mice, which accept human cells without irradiation. We treated the mice with VAP-1 inhibitor LJP1586 or PBS (control) for 6 weeks as schematically presented in Fig. 5a. When analyzing the presence of human HSC (mCD45^−^hCD45^+^hCD34^+^hCD38^−^) in BM, the frequency of CD34^+^CD38^−^ cells (gate P-4) increased by 63% (2.29 vs. 1.4, *p* = 0.014) and the frequency of CD34^+^CD38 ^+^cells (gate P-5) also showed a trend of increase (15.31 vs. 12.74, trend: *p* = 0.09) in LJP-1586 treated mice (Fig. 5b, c).

For additional in vivo engraftment studies, we isolated two BM HSC pools by sorting: one containing only VAP-1^−^ HSC and one containing 14,5% VAP-1^+^ HSC (the rest were VAP-1^−^ in this pool, since the low numbers of VAP-1^+^ HSC made it technically impossible to use a pool containing only VAP-1^+^HSC). The cells were adoptively transferred to NBSGW mice (Fig. 5d, e). After the transfer, the mice were treated with VAP-1 inhibitor LJP-1586 for 6 wks or left untreated. None of mice transplanted with VAP-1^−^ HSC demonstrated engraftment. In contrast, 3/3 mice having VAP-1^+^ cells in the transfer pool and receiving the VAP-1 inhibitor accepted the human BM engraftment (Fig. 5f). These data collectively suggest that while VAP-1^+^ HSC identifies the proliferating HSC pool, inhibition of VAP-1 activity allows higher than physiological rate of HSC engraftment and expansion. This supports the idea that VAP-1 is a check-point inhibitor regulating HSC proliferation.

### The number of HSC and HSPC is increased in the BM of VAP-1-KO and VAP-1-KI mice

**Fig. 6:**
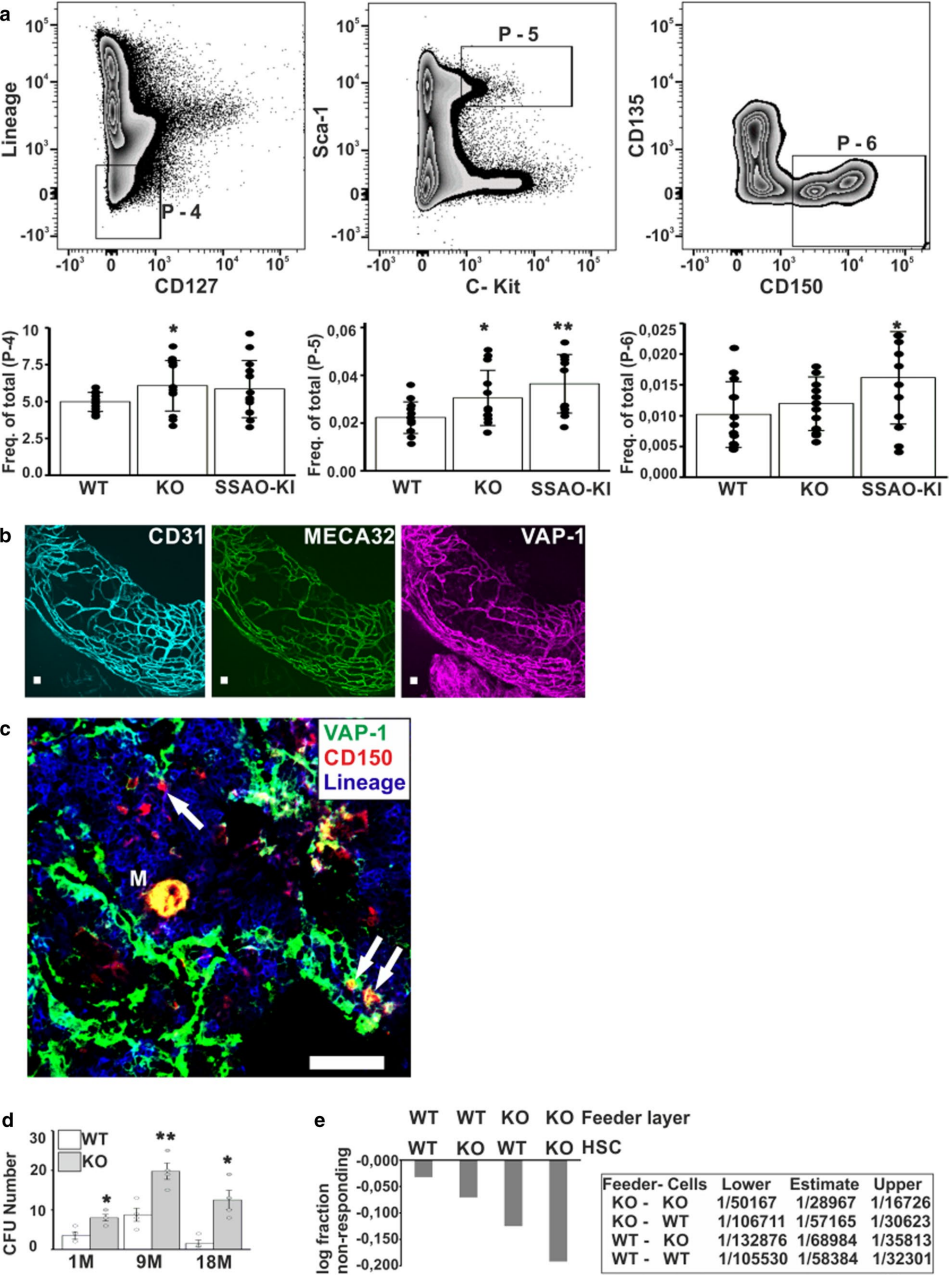
The number of primitive HSC is increased in BM of VAP-1-deficient mice. **a** BM cells of WT, VAP-1-KO, and VAP-1-KI mice were stained with Lineage cocktail, anti-CD127, anti-c-Kit anti-Sca-1, anti-CD135, and anti-CD150 antibodies and analyzed by flow cytometry. The plots show the gating strategy for HSCs. While gates P-1, P-2, and P-3 (not shown) exclude debris and doublets, gates P-4, P-5, and P-6 show the sequential enrichment of HSC, with gate P-6 representing the purest population. The columns show the relative frequency of gated cells among total nucleated cells isolated from WT, KO and SSAO-KI mice (*n* = 5). Student’s t-test was applied (*p-value < 0.05, **p-value < 0.01). **b** Whole-mount immunofluorescence staining of a mouse femur for VAP-1, MECA-32, and PECAM-1/CD31. VAP-1 is present in all vessels (representative of 10 mice). **c** Immunofluorescence staining of Lineage-CD150 + HSC and VAP-1-expressing blood vessels (green). Arrows indicate HSC interacting with VAP-1-expressing blood vessels. M indicates a megakaryocyte expressing CD150 and VAP-1 (representative of 5 mice). Scale bars represent 50 μm. **d** HSC among BM cells. The numbers of CFUs formed following seeding of 2500 BM cells isolated from young (1-month-old, 1 M), adult (9-month-old, 9 M), and very old (18-month-old, 18 M) mice are shown (*n* = 4). Student t-test was applied (*p-value < 0.05, **p-value < 0.01). **e** The LTC-IC potential of primitive progenitors among BM cells. The frequencies were determined by culturing 12′500 BM cells on irradiated BM-derived feeder layers for 4 weeks. Four different combinations were used by culturing HSCs derived from WT or VAP-1-KO mice on feeder layers derived from WT or VAP-1-KO mice. The table provides a summary of the frequency of HSC in culture from 6′225, 12′500, and 25′000 starting BM cells (feeder layer from 3 mice, BM HSC from 5 mice). L-cal software was applied

Next, we investigated, whether a lack of VAP-1 affects the number of HSPC and HSC in the mouse BM. To this end, we performed flow cytometric analysis of HSPCs and HSCs derived from the BM of VAP-1-KO, VAP-1-KI (having the VAP-1 protein without enzymatic activity), and WT mice. The number of HSCs, defined as Lineage^−^Sca-1^+^c-Kit^+^CD150^+^CD127^−^CD135^−^ cells, was significantly higher in the BM of VAP-1-KO and VAP-1-KI mice than in the BM of WT mice (0.013% n = 5, 0.017% n = 5, and 0.011% n = 5, respectively, gate P-6 in Fig. 6a). Sequential staining with HSPC markers showed that Lineage^−^CD127^−^ cells (gate P-4) and c-Kit^+^Sca-1^+^ cells (gate P-5) were enriched in VAP-1-KO mice (within both gates) and VAP-1-KI mice (within gate 5). However, unlike in human, mouse HSC were negative for VAP-1 (Fig. S9a). In fact, VAP-1 colocalized with the endothelial cell markers, CD31 and MECA-32 (recognizing PLVAP) in the vast majority of arterioles and most microvessels (Fig. 6b). Moreover, lineage^−^CD150^+^ cells, which include HSPC among other cell types [56] were largely located in close proximity to, and likely attached to, VAP-1-expressing blood vessels (Fig. 6c) suggesting that VAP-1 is part of the hematopoietic niche containing HSPC that is destroyed by irradiation(fig. S9b).

### Colony forming capacity of BM cells is increased in VAP-1 KO mice

We next used VAP-1-KO mice for functional tests by performing short-term CFU assays with BM cells harvested from VAP-1-KO mice aged 1, 9, and 18 months. The total colony count included progeny from HSC, multipotent progenitors, and lineage-committed progenitors. The number of CFUs was much higher using BM cells isolated from VAP-1-KO mice than using BM cells isolated from WT mice at all ages, but the difference was at highest using cells isolated from 18-month-old mice (eightfold, *n* = 4 in each age matched group; Fig. 6d). We also expanded BM-derived HSC and multipotent progenitors (HSPC) on a BM-derived feeder layer that had been irradiated [57, 58]. CFU assays demonstrated that expansion was increased by ~ twofold when HSPC derived from VAP-1-KO mice were grown on a VAP-1-KO feeder layer in comparison to cultures on WT feeders (Fig. 6e). Expansion was reduced when HSC and HSPC derived from VAP-1-KO mice (*n* = 5) were grown on a WT feeder layer and when HSC and HSPC derived from WT mice (*n* = 5) were grown on a VAP-1-KO feeder layer (pooled from BM of three KO and three WT mice). In conclusion, VAP-1 expressed by stromal cells markedly affects the proliferation of HSC and HSPC. Based on these experiments, we conclude that VAP-1 is important for maintenance of HSC and HSPC. The number of HSC and HSPC was increased in VAP-1-KO mice, and the findings made in VAP-1-KI mice indicate that this effect was due to the lack of VAP-1 enzymatic activity.

### VAP-1 inhibitor increases the numbers of HSC

**Fig. 7:**
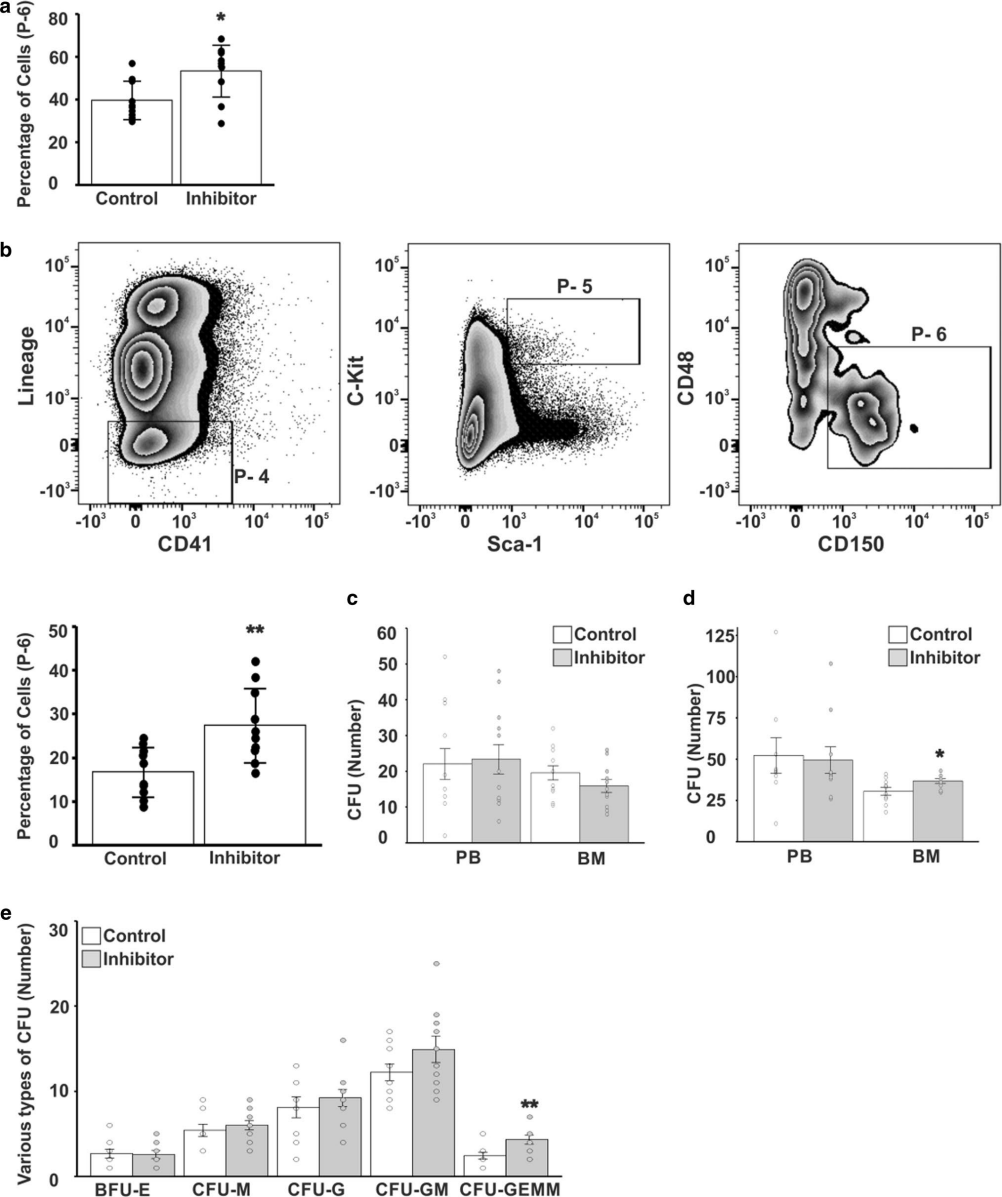
LJP-1586 treatment increases the number of HSC in BM of mice. **a** Percentages of HSC within gate 6 (shown in Fig. 6a) in the groups treated by inhibitor LJP-1586 and control PBS (*p*-value < 0.05). **b** FACS profiles showing gradual enrichment of HSC using a different set of HSC markers as used in Fig. 2a (Lin-CD41-Sca-1 + c-Kit + CD48-CD150 +) and the bars show the percentage of HSC from gate P-6 (*p*-value < 0.01). **c** CFU assay using peripheral blood (PB) or BM cells isolated after the 7-day treatment with LJP-1586 (*n* = 4). **d** CFU assay using PB or BM cells isolated after the 42-day treatment with LJP-1586 (*n* = 10) (*p*-value < 0.05). **e** Analysis of BM derived subtypes of CFUs shown in **d**. *BFU-E *Burst forming erythroid unit, *CFUM *macrophage colony forming unit, *CFU-G *granulocyte colony forming unit, *CFUGM *granulocyte/macrophage colony forming unit, *CFU-GEMM* granulocyte/erythroid/macrophage/megakaryocyte colony forming unit. Student *t*-test was applied (*p*-value < 0.01)

To confirm that proliferation of HSC is increased in VAP-1-deficient mice, we injected 2 weeks old WT mice with the small molecule LJP-1586, which blocks the enzymatic activity of VAP-1. The treatment was continued for 6 weeks. HSC in BM were then analyzed using the same markers as in Fig. 6a (defined as Lineage^−^Sca-1^+^c-Kit^+^CD150^+^CD127^−^CD135^−^ cells). The percentage of HSC (gate 6 in Fig. 6a) was 34% (PBS, *n* = 10; LJP-1586, *n* = 10) higher in LJP-1586-treated mice than in control mice (Fig. 7a). When we defined HSC as Lineage^−^Sca-1^+^c-Kit^+^CD150^+^CD48^−^CD41^−^ cells [59–62], the percentage of these cells was increased by 65% in LJP-1586-treated mice (Fig. 7b). These results demonstrate that the number of HSC is increased in mice treated with LJP-1586, irrespective of which markers are used to define HSC.

We also performed CFU assays to study the effects of VAP-1 inhibition on colony formation. Upon culture in methylcellulose-based medium, the number of CFUs formed by BM cells isolated from mice treated with LJP-1586 (*n* = 4) for 1 week and non-treated control (*n* = 4) mice did not significantly differ (Fig. 7c). This indicates that mice must be treated with LJP-1586 for a longer duration to elicit effects on stem cell expansion. When the duration of treatment was increased to 6 weeks (*n* = 10 + 10), the number of CFUs formed by BM cells isolated from LJP-1586-treated mice was 20% higher than the number of CFUs formed by BM cells isolated from the control mice (36.7 vs. 30.5 colonies, *p* = 0.0396; Fig. 7d). By contrast, the number of CFUs formed by peripheral blood (PB) cells did not significantly differ between LJP-1586-treated and control mice (49.4 vs. 52.2 colonies, *p* = 0.8373). Differential counts of the BM cell-derived colonies revealed that the number of CFU-GMs was increased by 22% (14.9 vs. 12.2, *p* = 0.0729) and the number of CFU-GEMMs was increased by 79% (4.3 vs. 2.4, *p* = 0.0459) in LJP-1586-treated mice. However, only the latter reached statistical significance (Fig. 7e). In summary, inhibition of the enzymatic activity of VAP-1 in mice increases the number of HSC, leading to the generation of more CFU-GEMMs.

## Discussion

Here we show that VAP-1 protein expression defines a novel HSC subset in humans. VAP-1^+^ HSC are genuine undifferentiated HSC which are transcriptionally distinct from the other HSC. VAP-1^+^ HSC closely resemble a novel HSC subpopulation, HSC2, which we identified from scRNAseq data. We show that HSC2 are proliferating and developmentally more mature than the other HSC. We also demonstrate that VAP-1 deficiency and inhibition of VAP-1 via a chemical inhibitor increases HSC proliferation and engraftment in vivo. Collectively these data suggest that VAP-1 is a new check-point inhibitor, which is induced on HSC before they undergo further differentiation.

mRNA signal of VAP-1 in HSC, including HSC2, was very low. This is not unexpected, since many key protein markers for HSC including CD90 is expressed at a notably low mRNA levels in HSC: This may be due to a slow turnover of the message [63]. Alternatively, soluble VAP-1 protein produced by other cells and found in the blood of healthy persons may bind to the surface of HSC. In either case, our data show that VAP-1 protein on HSC surface identifies a unique subpopulation of HSC.

The enzymatic activity of VAP-1 leads to production of ROS, which influence the development and self-renewal of HSC [64]. Low levels of ROS are required for maintenance of HSC [65], and intermediate levels of ROS drive proliferation and differentiation [64, 66], while high levels of ROS lead to damage and exhaustion of the stem cell pool [22, 23]. As an important but not the sole source of ROS, VAP-1 may help to fine-tune the ROS concentration. Other enzymes, apart from SSAOs, do not produce large amounts of H2O2 in HSC [67]. Our results from RNAseq analyses of BM VAP-1^+^ and VAP^−^ HSC together with the findings that the p53 signaling pathway was highly enriched among VAP-1^+^ cells and VAP-1 inhibitors decreased ROS production suggest that VAP-1 created ROS at least partially act via the p53 signaling pathway.

When we took advantage of the existing single cell sequencing data from human BM HSC [10] and analyzed its possible heterogeneity, we could identify two clearly distinct populations, which we named HSC1 and HSC2. Further analyses showed that the genes highly expressed in VAP-1^+^ HSC, including *CDC8A*, and *TPX2*, were present almost exclusively in the HSC2 population. Similarly, sorted VAP-1^+^ HSC expressed HSC2 signature genes (*TYMS*, *PCNA*) at significantly higher levels than did VAP-1^−^ HSC. Additionally, comparison of four DEG lists together unambiguously showed that VAP-1^+^ HSC and HSC2 share common GO terms and they cluster together. Therefore, our data suggest that VAP-1^+^ HSC and HSC2 are largely overlapping HSC populations. Notably, VAP-1 protein expression should thus be a useful marker allowing prospective enrichment of HSC2 cells for further studies.

The VAP-1^+^/HSC2 population shows characteristics of proliferating cells. On the other hand, we found that inhibition of VAP-1 increases proliferation and engraftment of HSC. The inhibitor experiments support a concept that high levels of ROS produced by VAP-1 inhibit HSC proliferation, while the low levels of ROS are supportive for HSC production. In this context it should be noted that chemical VAP-1 inhibition regimens used lead to reduced and fluctuating VAP-1 activity rather than its complete abrogation. We suggest that VAP-1 is a new check-point inhibitor controlling HSC proliferation. In this scenario, VAP-1 will be induced on HSC surface when the cells leave dormancy and move towards a proliferative state, i.e. when HSC1 differentiate into HSC2. On HSC2 cells VAP-1 activity then starts to inhibit proliferation, possibly to maintain the stem cell nature of the HSC. If VAP-1 is inhibited at this stage, the outcome is enhanced, supra-physiological proliferation. In this manner, VAP-1 on HSC would function analogously to immune check-point inhibitors on T-cells. These molecules, including CTLA-4 and PD-1, are induced on activated T-cells and serve as intrinsic brakes for T-cell proliferation and differentiation during an immune response, and inhibition of check-point inhibitors on T-cells with drugs leads to enhanced immune reactions [68, 69].

Unexpectedly, mouse HSC were found to be negative for VAP-1. Since the in vivo experiments utilizing VAP-1 KO mice and VAP-1 inhibitor treatments unambiguously demonstrated the role of VAP-1 in HSC proliferation (increase in HSC and CFU-GEMM numbers), the effect has to come from vascular VAP-1 in mice. Lineage^−^CD150^+^ cells largely resided close to VAP-1-expressing blood vessels. This is not unexpected because the close proximity of HSC to blood vessels has already been reported and the significance of the vascular niche has become evident [52, 56]. However, the localization of VAP-1 may mean it can contribute to the HSC niche via direct contact with HSC by altering the microenvironment via its enzymatic activity producing ROS. The possibility for direct binding of HSC to vascular VAP-1 also exists, as the recent single cell sequencing finds Siglec-10 message among HSC [10]. Siglec-10, on the other hand, has been shown to be a leukocyte ligand for VAP-1 [70]. BM irradiation dramatically destroys BM sinusoids, which provide the niche for about 80% of dividing and non-dividing HSC [71–73]. We also witnessed the loss of VAP-1 in BM vasculature subsequent to irradiation preventing to study VAP-1 dependent engraftment of transplanted cells after irradiation.

In conclusion, we define VAP-1 as a cell surface marker for a novel proliferating HSC subpopulation. Mechanistically VAP-1 serves as a check point-like inhibitor restricting the proliferation of HSC in a manner which at least partially involves VAP-1 dependent regulation of ROS levels. Notably, VAP-1 is readily targetable with small molecule inhibitors. In fact, different VAP-1 inhibitors have been and currently are in clinical trials for inflammatory diseases and have not caused any safety concerns (http://www.pharmaxis.com.au/investor-centre/news/view/pharmaxis-releases-successful-results-of-phase-1-clinical-trial-for-boehringer-ingelheim-partnered-drug-pxs4728a and https://www.astellas.com/en/ir/ar2017/pdf/2017AR_53_en.pdf). Based on our findings, they may be therapeutically exploitable also in HSC expansion.

## Supplementary Information

Below is the link to the electronic supplementary material.

Supplementary file1 (XLSX 13 KB)

Supplementary file2 (XLSX 4734 KB)

Supplementary file3 (XLS 40 KB)

Supplementary file4 (XLSX 382 KB)

Supplementary file5 (DOCX 3089 KB)

## Data Availability

The bulk and single cell RNA-seq data have been deposited to the Gene Expression Omnibus with SRA accession number PRJNA594799 and PRJNA729883 respectively. Further information and requests for unique reagents used in this study should be directed to and are available without restrictions from the Lead Contact, Sirpa Jalkanen (sirjal@utu.fi).

## Code availability

Consents to participate and for publication N/A.
