# Supplementary material for: Vascular adhesion protein-1 defines a unique subpopulation of human hematopoietic stem cells and regulates their proliferation
Source: Cell Mol Life Sci. 2021 Nov 1;78(23):7851–72. doi: 10.1007/s00018-021-03977-6 (PMC8629906; doi:10.1007/s00018-021-03977-6)
Supplement: Supplementary file 5 — Supplementary file5 (DOCX 3089 KB) [file 18_2021_3977_MOESM5_ESM.docx]

**Vascular adhesion protein-1 defines a unique subpopulation of human hematopoietic stem cells and regulates their proliferation**

**Imtiaz Iftakhar-e-Khuda^1^, Alberto Pessia^2^, Shuyu Zheng^2^, Matti Kankainen^2^, Mika Kontro ^3^, Marika** **Karikoski^1^, Sina Tadayon^1^, Maija Hollmén^1^, Jing Tang^2^, Beat A. Imhof^1,4^, Marko Salmi^1^ and** **Sirpa Jalkanen^1*^**

* Corresponding author. Email: sirjal@utu.fi

**Supplementary Figures**


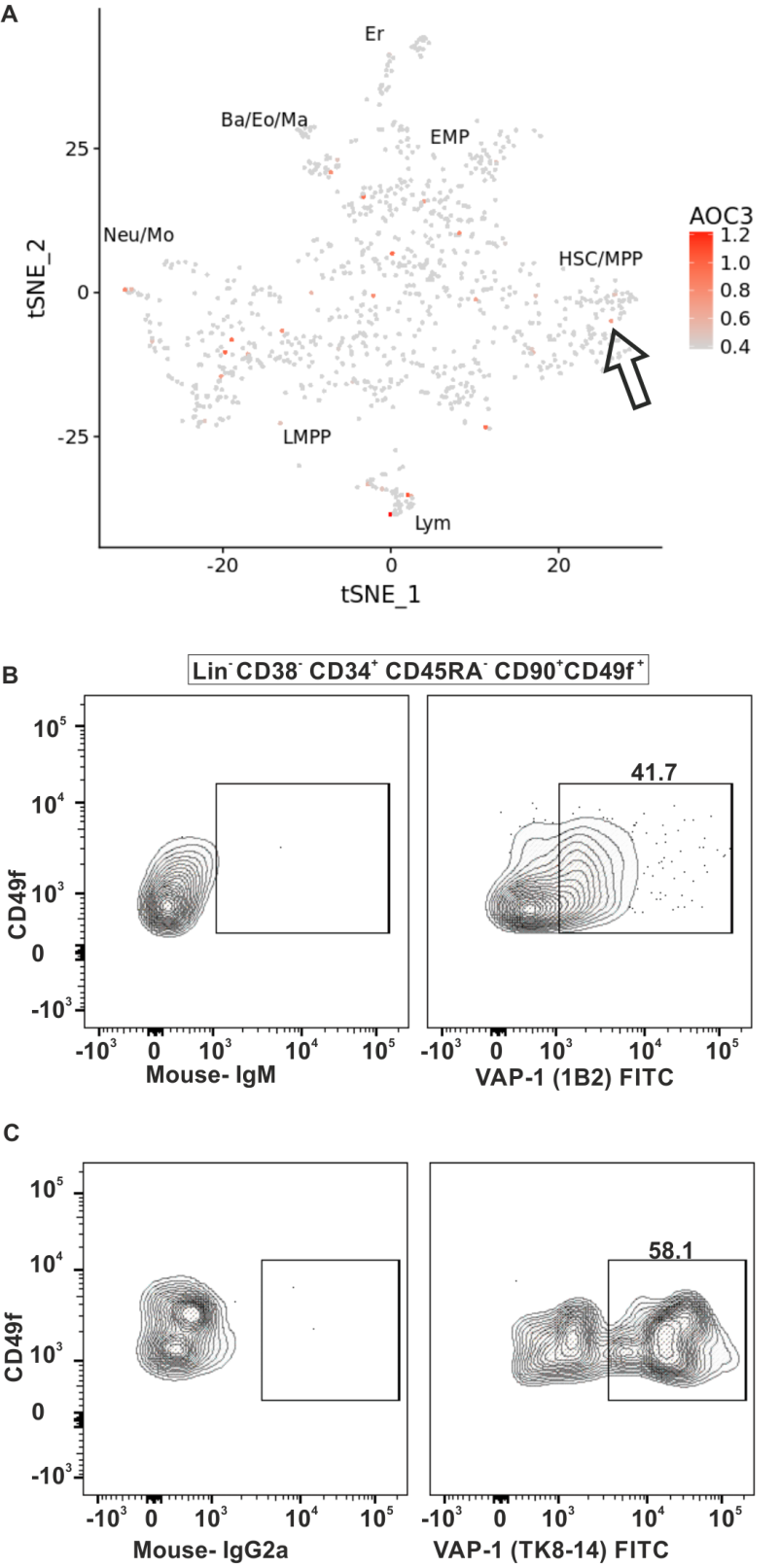


**Fig S1** **VAP-1 expression on HSC**. **a** *AOC3* expression on CB CD34^+^ enriched cells. The Figure is collected from online resource (<http://satijalab.org/cd34/>). **b** and **c** Detection of surface expression of VAP-1 on CB-derived HSCs using anti-human VAP-1 antibodies (1B2 and TK8-14) and isotype-matched controls. Representative of ten samples.


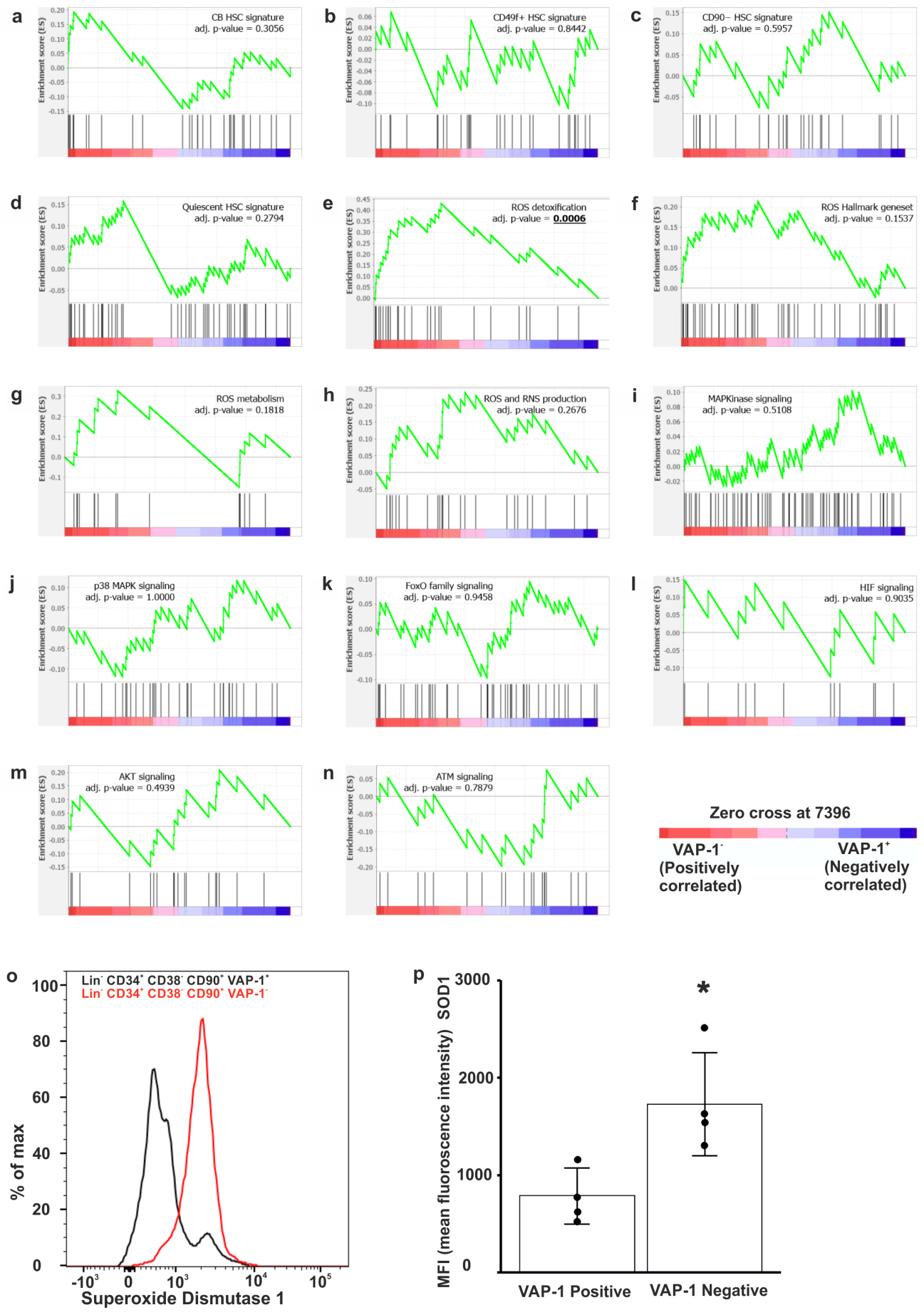


**Fig S2 Pathway analyses of VAP-1^+^ HSC. a** GSE analyses of cord blood (CB) HSC. **b** CD49f^+^ cells, **c** CD90^-^ cells, **d** quiescent HSC, **e** ROS detoxification, **f** ROS hallmark, **g** ROS metabolism, **h** ROS and RNI production, **i** MAPkinase signaling, **j** p38 signaling, **k** FOXO signaling pathway, **l** HIF signaling, **m** AKT signaling, and **n** ATM signaling signatures. Note that only the ROS detoxification signature is significantly correlated to VAP-1^+^ cells; FDR, false discovery rate. Input gene sets for GSE analyses are shown in Supplementary information Table S4. **o** Superoxide dismutase 1 protein expression is detected on pooled cord blood VAP-1 positive and negative cells using FACS. **p** Superoxide dismutase 1; MFI calculated from pooled samples, n=4 (p-value < 0.05)


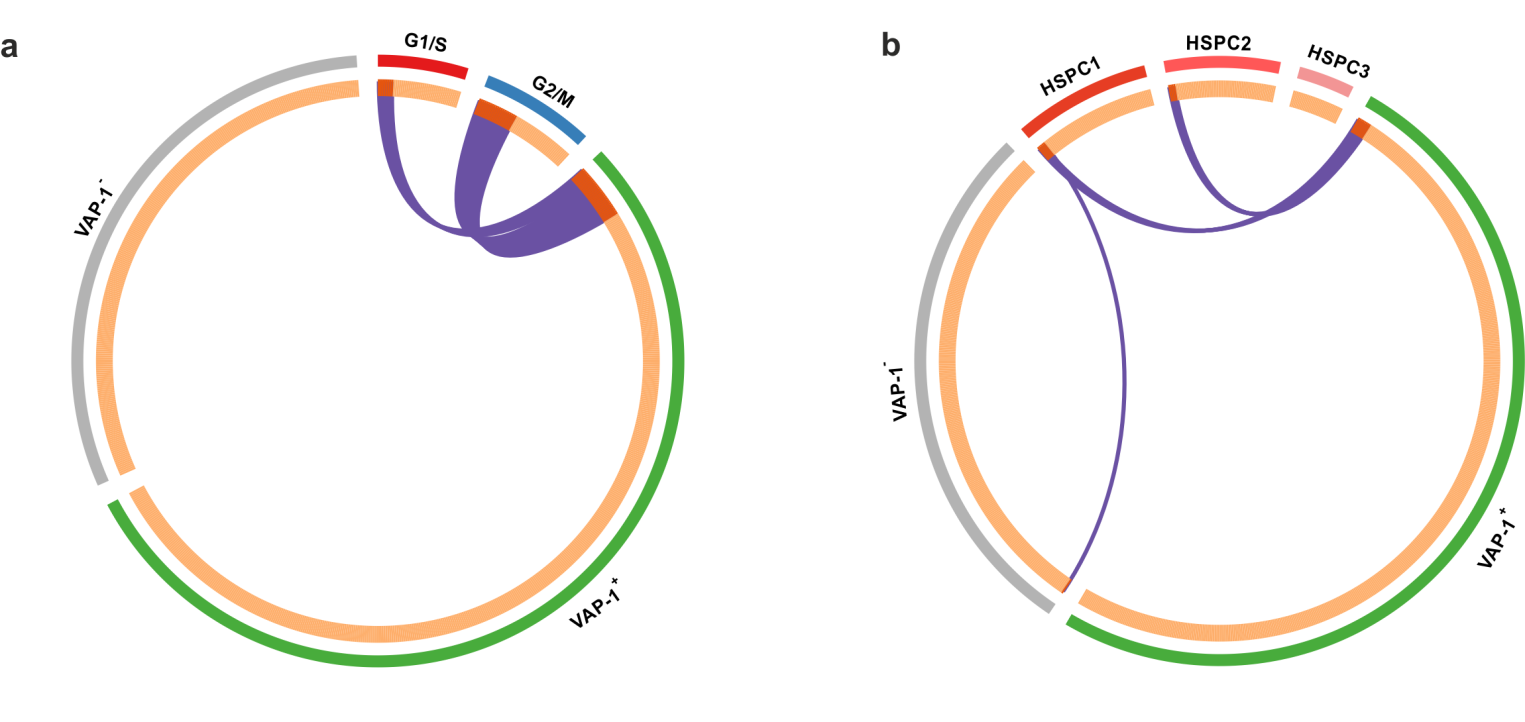


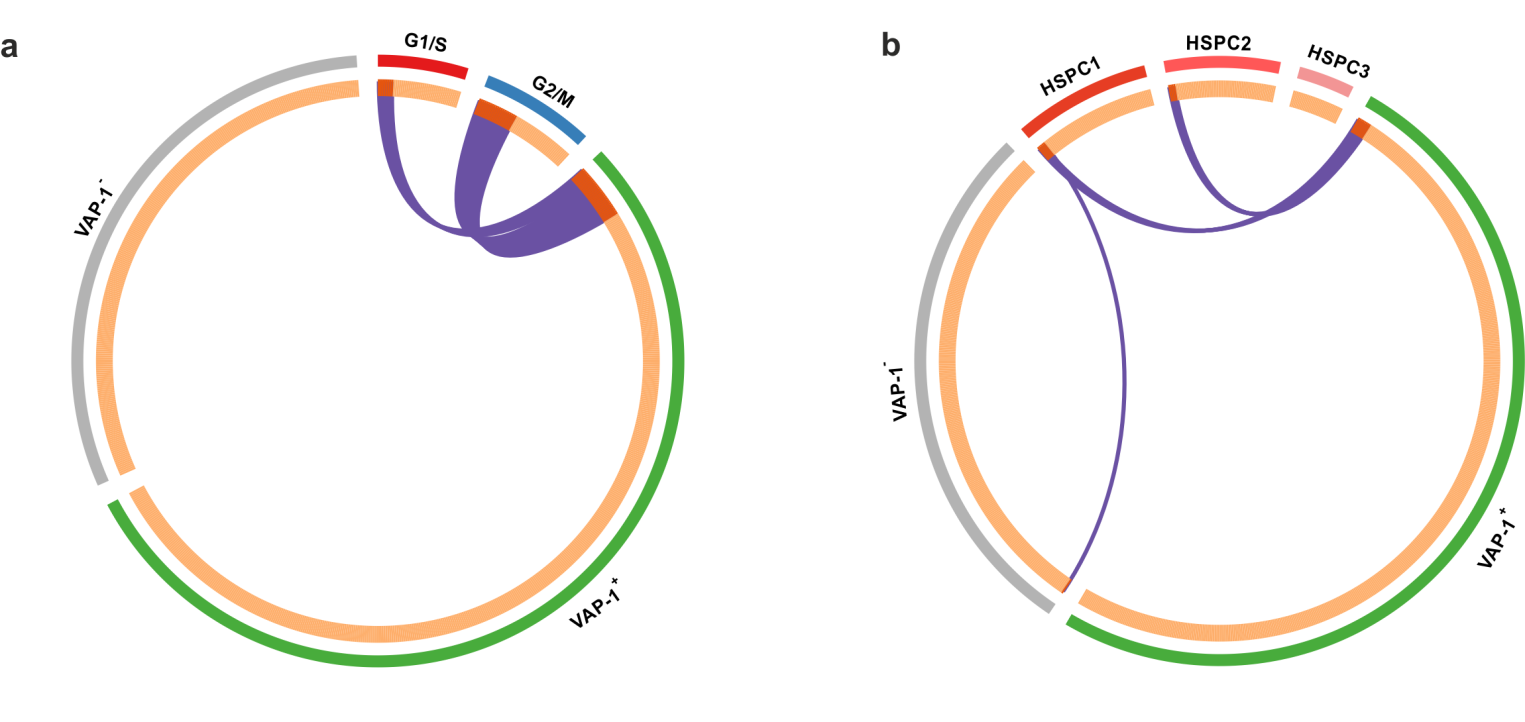


**Fig S3 VAP-1^+^ cells in adult human BM are proliferative potential containing HSC**. **a** Meta-analysis showing G1/S and  G2/M genes enriched among the top differentially expressed genes of VAP-1^+^ cells. **b** HSPC1 and HSPC2 genes enriched among the top differentially expressed genes of VAP-1^+^ cells.


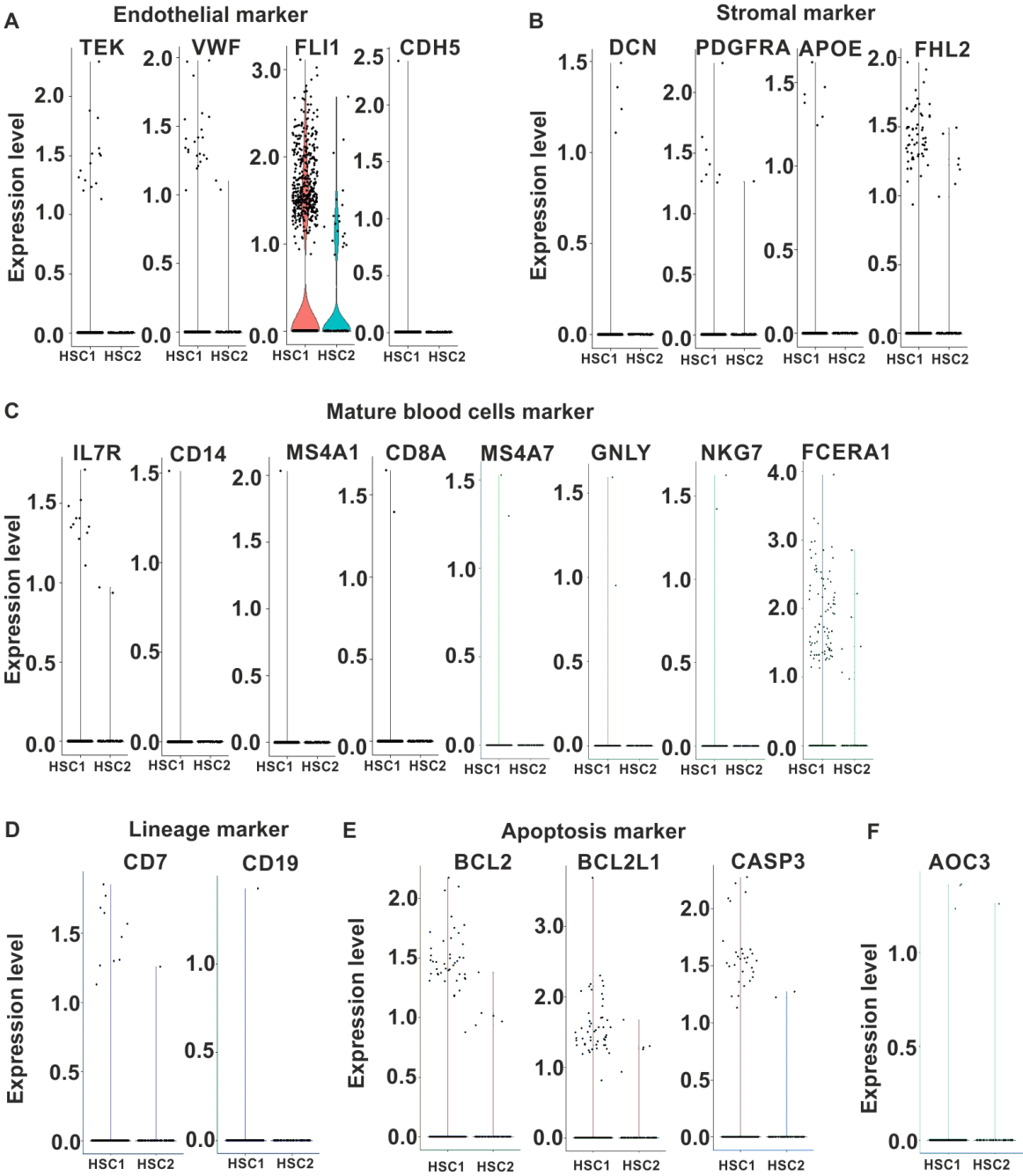


**Fig S4 Distribution of lineage and apoptosis markers and *AOC3* in HSC1 and HSC2 clusters**. Violin plots showing expression of human **a** endothelial, **b** stromal, **c** mature blood cells, **d** lineage and **e** apoptosis markers and **f** VAP-1 encoding gene *AOC3* in the indicated clusters (from the scRNAseq data).


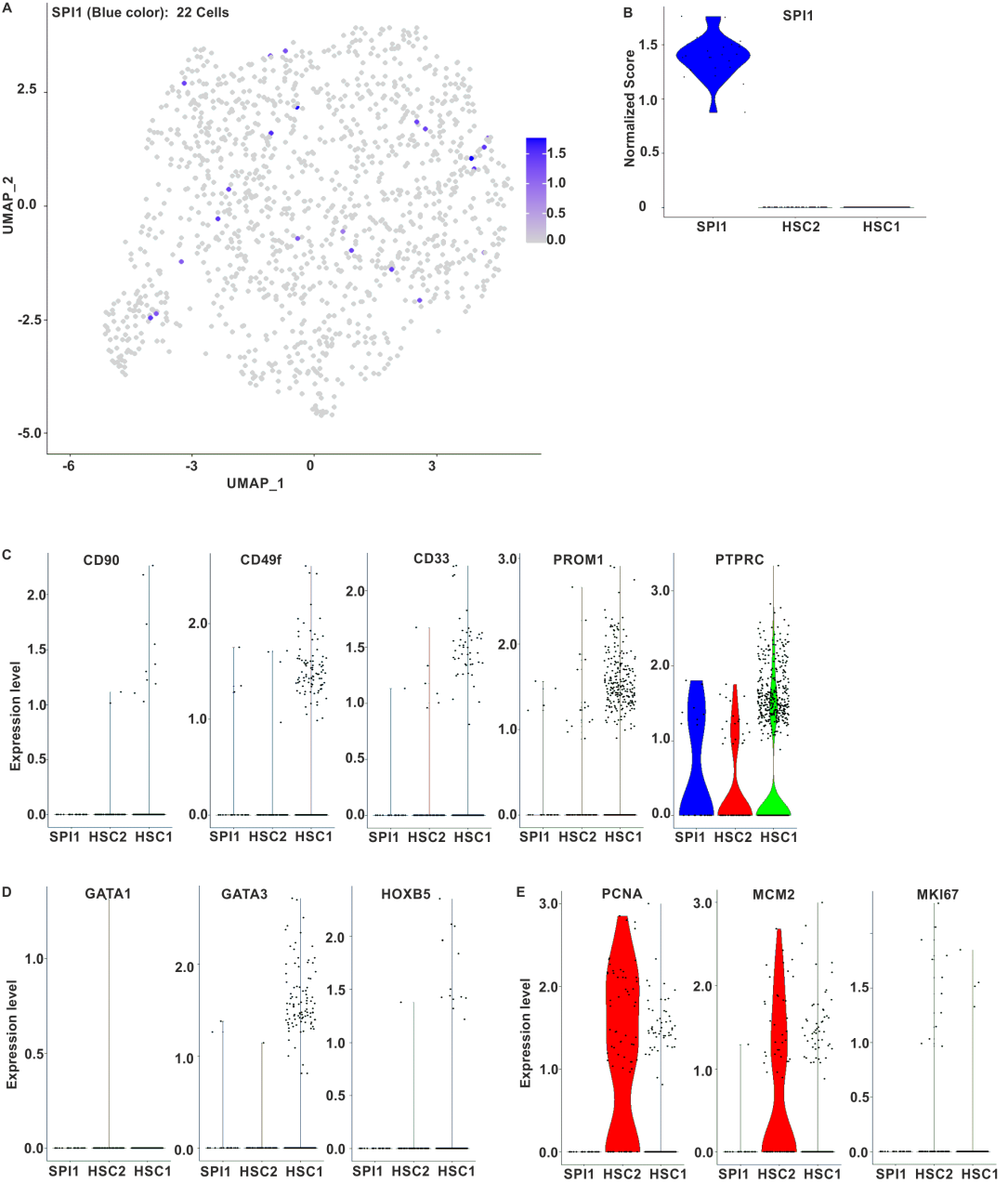


**Fig S5 Analysis of *SPI1* expressing HSC in human BM.** Analysis of *SPI1* expressing HSC in human BM. **a** Analysis of *SPI1* expressing HSC (blue cells), which are dispersed within the HSC1 and HSC2 clusters. **b** *SPI1*^+^ cells (22 events) are distinct from HSC1 and HSC2 clusters. **c** Expression scores of selected HSC surface markers, **d** known HSC-related transcription factors and **e** signature genes for cell proliferation in the indicated HSC clusters.


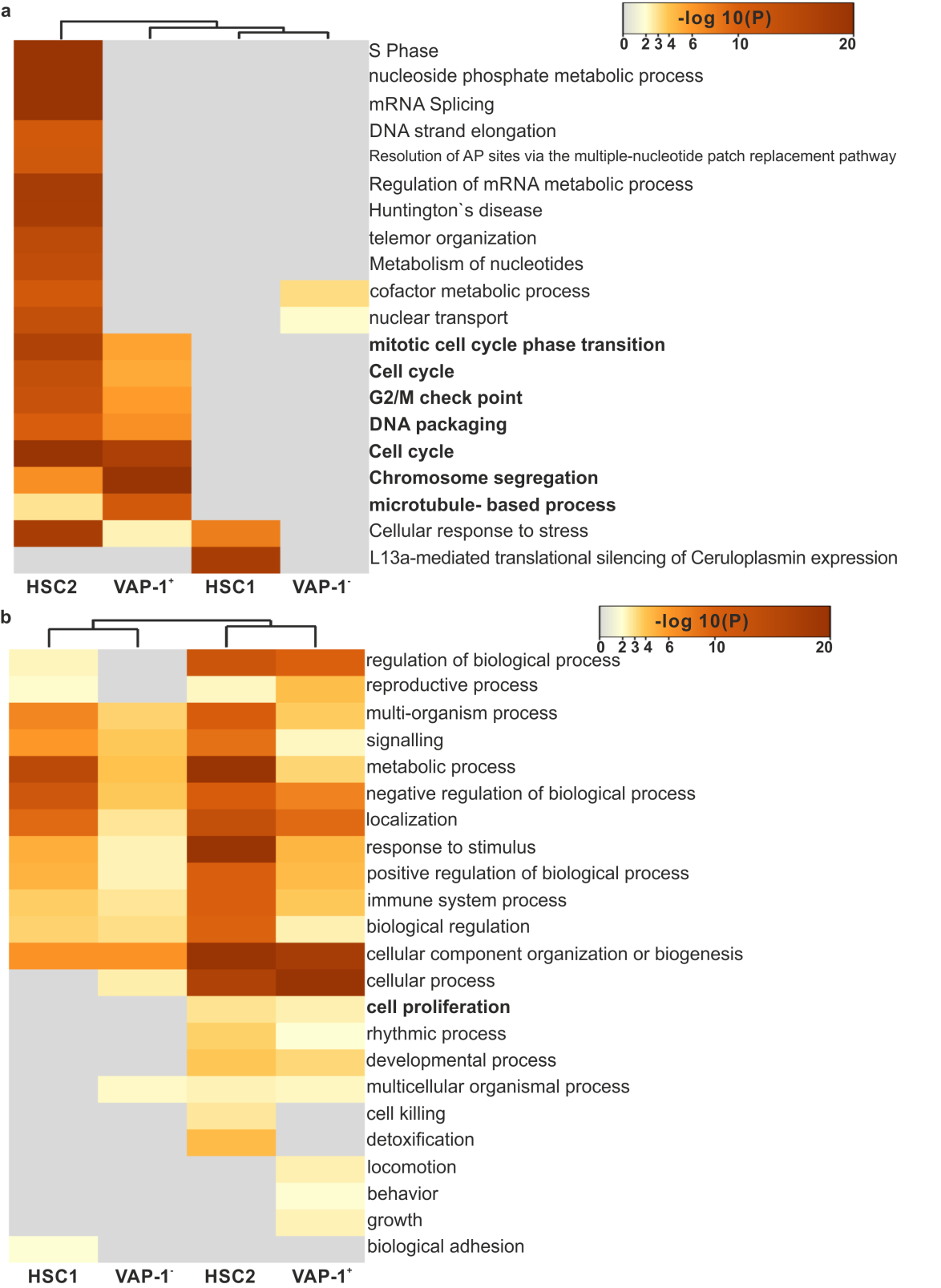


**Fig S6 Combined pathway analyses of VAP-1^+^ and HSC2**. **a** Gene ontology: enrichment analyses of pathways and **b** biological processes of differentially expressed genes from VAP-1^+^, VAP-1^-^ , HSC1 and HSC2 data sets.


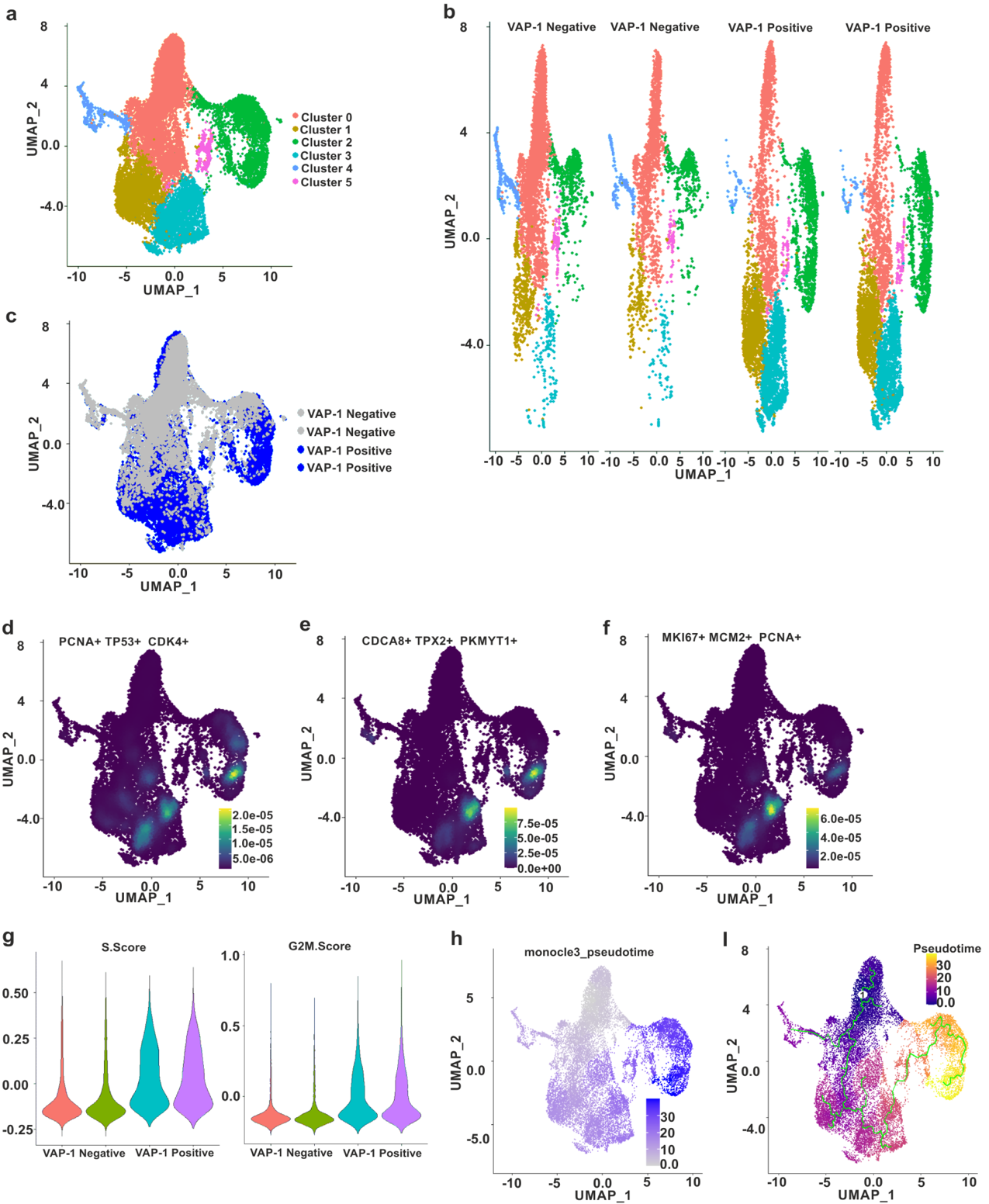


**Fig S7 VAP-1^-^ and VAP-1^+^ HSC from adult human BM have different cell proliferation scores. a** UMAP projection of six hematopoietic stem cell groups among VAP-1 positive (n=2) and VAP-1 negative(n=2). **b** UMAP projection of all samples separately. **c** UMAP projection of VAP-1 positive and negative sample groups. (**d-f)** With nebulosa’s kernal function utilising low dimension (UMAP) cellular density features cells ordered based on p53 pathways (d), top genes identified in bulk RNAseq (**e**) and (**f**) cell proliferation signature genes identified in bioinformatics analysis of Pellin’s data (<https://github.com/powellgenomicslab/Nebulosa>). **g** Expression of S.Score and G2M.Score in each sample group after linear and non-linear dimensional reduction.(**h-i)** UMAP plot of the pseudotime (h) and developmental (**i**) trajectory generated using Monocle 3 (<https://cole-trapnell-lab.github.io/monocle3/>). Monocle3 estimates with Cluster 0 as the root showing the inferred differentiation.


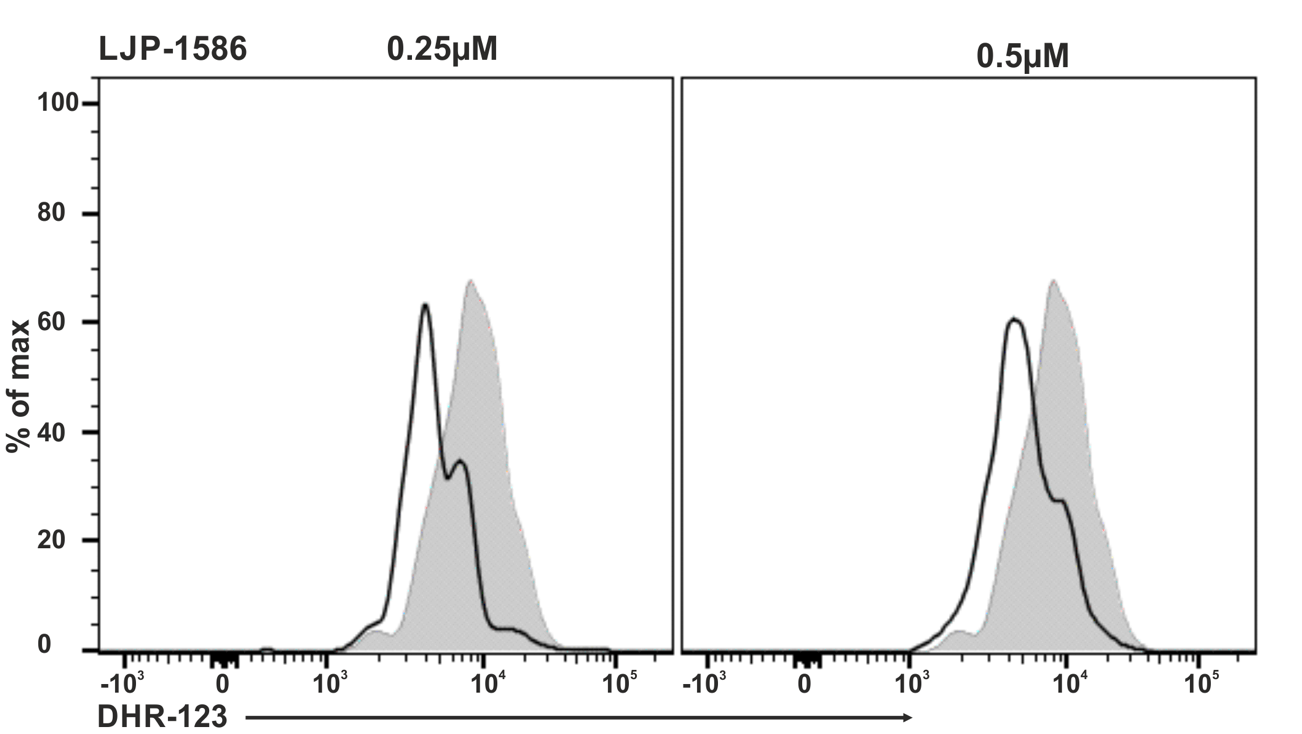


**Fig S8** **VAP-1 inhibition reduces ROS production of HSC in liquid cultures**. ROS were detected by DHR- 123 in living PMA-stimulated CD38^-^, CD34^+^ gated BM HSC after 9-day liquid cultures containing the indicated concentrations of LJP-1586. Closed histograms show control conditions, open histograms represent HSC cultured in presence of LJP-1586, (one donor with two technical repeats).


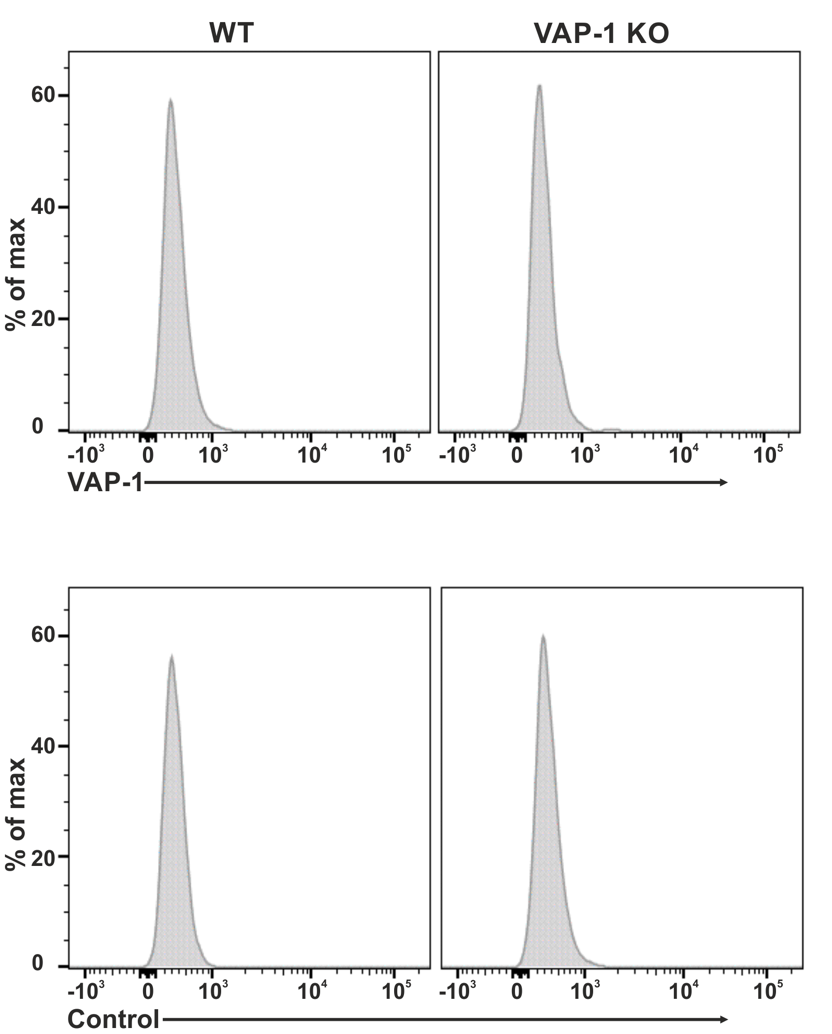


**Fig S9 Absence of VAP-1 expression on mouse BM derived HSC**. The gating P-5 of enriched HSC (Lin-Sca-1+c-Kit+) is shown in Fig. 2a. A mixture of two anti-VAP-1 antibodies (polyclonal antibody and monoclonal 7-106 antibody) was used to detect surface expression of VAP-1 on HSC from BM of WT and VAP-1-KO mice, isotype-matched antibodies were used as controls (rabbit IgG and rat IgG2b), n= 5.
